# Machine learning prediction and calibration of cellulose-based solid-phase extraction performance for pharmaceuticals across aqueous matrices

**DOI:** 10.1039/d5ra09776b

**Published:** 2026-03-04

**Authors:** Ephriam Akor, Damilare Olorunnisola, Moses O. Alfred, Onome Ejeromedoghene, Martins O. Omorogie

**Affiliations:** a African Centre of Excellence for Water and Environmental Research (ACEWATER), Redeemer's University P.M.B 230 Ede Osun State 232101 Nigeria omorogiem@run.edu.ng dromorogiemoon@gmail.com; b Department of Chemical Sciences, Faculty of Natural Sciences, Redeemer's University P.M.B 230 Ede Osun State Nigeria; c Institute of Chemistry, University of Potsdam D-14476 Potsdam Germany; d University of Potsdam, Institute of Nutritional Science 14558 Nuthetal (Ortsteil Bergholz-Rehbrücke) Arthur-Scheunert-Allee 114-116 Germany; e State and Local Joint Engineering Laboratory for Novel Functional Polymeric Materials, College of Chemistry, Chemical Engineering and Materials Science, Soochow University Suzhou Jiangsu Province 215123 P. R. China

## Abstract

Cellulose-based solid-phase extraction has been increasingly proposed for concentrating trace pharmaceuticals from complex waters; however, cross-laboratory transfer remains uncertain because studies vary in matrix chemistry, sorbent functionalization, extraction format, elution strategy, and quality control. Evidence from 2015 to 2025 was gathered, and 637 experiments from 36 reports and 28 DOIs were modelled using 29 descriptors of method and matrix. ElasticNet (EN), XGBoost (XGB), and random forest regressor (RFR) were evaluated using study group nested cross-validation with conformal prediction to estimate out-of-study performance and 90% confidence intervals for recovery, matrix recovery ratio (MRR), enrichment factor (EF), limit of detection (LOD), and limit of quantification (LOQ). ElasticNet dominated the sensitivity endpoints, achieving a mean *R*^2^ of 0.99999 for the enrichment factor, 0.99985 for the limit of detection, and 0.99914 for the limit of quantification, with mean 90% interval widths of 0.300, 44.386, and 829.752, respectively. For the recovery and matrix recovery ratio, random forest has the strongest correlation but remained weakly predictive, with top settings yielding a mean *R*^2^ of about −0.52 and MAE of about 15.53 for the recovery and a mean *R*^2^ of about −1.03 and MAE of about 21.39 for the matrix recovery ratio, with 90% confidence intervals of 0.651, most pronounced for wastewater and river matrices. Decision maps were used to translate these contrasts into operating guidance and reporting priorities for matrix descriptors needed to support defensible local validation and method transfer.

## Abstract 2

Cellulose-based solid-phase extraction has been proposed for concentrating trace pharmaceuticals from complex waters; however, cross-laboratory transfer remains uncertain because studies vary in matrix chemistry, sorbent functionalization, extraction format and elution strategy.

## Graphical abstract

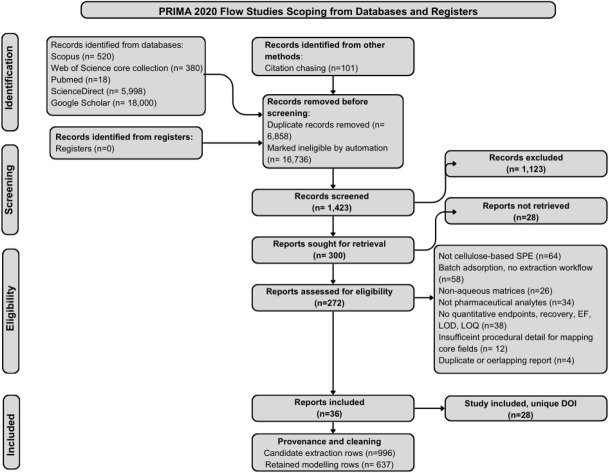

## 1 Introduction

The analyses of pharmaceuticals and personal care products have shifted from sporadic detections to routine, multi-class contamination across surface waters and engineered water cycles, with exposures now documented across diverse climates, catchment types, and sanitation infrastructures.^1,2^ Wilkinson and colleagues (2022) provided information on the quantification of active pharmaceutical ingredients of 1052 sampling sites across 258 rivers in 137 countries, demonstrating that analytical workflows must remain quantitatively stable across wide gradients in dissolved organic matter, salinity, and co-contaminants.^2^ Consistent with this global situation, Hernández Tenorio and colleagues (2022) gathered global evidence for 2018 and 2019 showed that concentrations commonly span ng per litre to microgrammes per litre across rivers, wastewater, lakes, groundwater, and drinking water, thereby requiring preconcentration, calibration, and matrix control to operate near the limits of routine quantitation.^1^

Based on the existing literature, solid-phase extraction remains a principal strategy for the enrichment and clean-up of trace organics, but comparative performance is often obscured by heterogeneity in sorbent chemistry, conditioning, elution composition, and quality control, despite repeated calls for systematic, mechanism-anchored method development.^3^ Matrix effects remain a dominant driver of error propagation in LC-MS/MS workflows, with suppression and enhancements capable of altering apparent recoveries and inflating between-study disagreement when internal standards, calibration models, and recovery correction practices are not harmonised.^4^ A recent direct injection monitoring study across 14 wastewater treatment plants illustrates the magnitude of the problem, reporting matrix effect values ranging from 8% to 190% in effluent wastewater, with recoveries ranging from near zero for some analytes to strong enhancement for others, reinforcing that matrix dependence is a first-order constraint for quantitative inference in complex waters.^5^

Renewed interest in cellulose-based sorbents is, therefore, scientifically and methodologically motivated since cellulose offers a modifiable surface that can express hydrogen bonding, electrostatic, and hydrophobic interactions while aligning with greener material choices and scalable fabrication routes.^6,7^ Olorunnisola and colleagues (2023) emphasised that cellulose can function as an unmodified phase or as an engineered derivative and composite for the extraction of pharmaceuticals and other polar organics, but they highlighted that performance evidence is fragmented across formats and laboratories, limiting transferable selection rules and discouraging robust cross-matrix decision support.^7^

Methodological gaps are increasingly visible at the interface of evidence synthesis and modelling, where adjacent porous material domains already use interpretable machine learning to map controllable process levers and predict functional properties. However, sample preparation and solid-phase extraction have adopted these approaches more cautiously and less consistently.^8,9^ Salamat and colleagues (2025) similarly note that artificial intelligence remains comparatively underused in extraction studies, particularly for uncertainty-aware decision support that can withstand cross-laboratory heterogeneity, incomplete covariate reporting, and matrix-specific failure modes.^10^ These limitations motivate a systematic, auditable synthesis approach grounded in contemporary reporting standards for evidence integration, since transparent study selection, variable harmonisation, and bias-aware inference are prerequisites for translating dispersed cellulose SPE performance claims into reliable, generalisable knowledge.^9^

Against this backdrop, the central question is no longer whether cellulose-based extraction can deliver acceptable recoveries in isolated demonstrations but whether the published evidence can be translated into transferable guidance that anticipates performance under new analyte and matrix conditions.^6,11^ Yadav and colleagues (2025) exemplify the current direction in related sorption research by curating literature data on biochar adsorption, benchmarking multiple algorithms, and using driver analysis to convert dispersed experiments into actionable design levers.^12^ Hassan and colleagues (2025) similarly show how ensemble modelling and SHAP-based explanation can formalise sensitivity to operating conditions and material properties for heavy metal sorption, shifting interpretation from narrative mechanisms to quantified influence patterns.^13^ Li and colleagues (2024) extend this logic to environmental remediation contexts by compiling evidence across heterogeneous experimental settings and using cross-validated models to infer which covariates govern adsorption effectiveness.^14^ In contrast, recent analytical reviews argue that AI adoption in sample preparation remains comparatively uneven, and that extraction studies often lack study-aware validation and the uncertainty quantification needed for trustworthy method transfer across laboratories and matrices.^15^

This study, therefore, integrates systematic evidence synthesis with study-aware predictive modelling for the cellulose-based solid-phase extraction of pharmaceuticals from aqueous matrices. The aim is to connect controllable method choices, sorbent and elution descriptors, analyte characteristics, and reported matrix context to the validation endpoints that govern analytical fitness for purpose, including recovery and matrix dependence, enrichment behaviour, and sensitivity measures, without collapsing the between-study structure that defines real-world transportability. The motivating questions concern whether recovery and sensitivity can be predicted from what is routinely reported, which variable families carry the most explanatory power once study heterogeneity is respected, and how uncertainty should be represented when deploying evidence beyond the source studies. This scope prioritises cellulose and cellulose-derived sorbents within aqueous monitoring workflows, because these systems sit at the intersection of green materials innovation and the practical constraint that pharmaceuticals are frequently present near method limits in chemically diverse waters.

This contribution is framed as a community-facing resource rather than a single model claim. A transparent synthesis protocol and harmonised variable dictionary are presented to maximise traceability and reuse, aligned with contemporary systematic review reporting expectations.^9^ Uncertainty is treated as an operational output through distribution-free predictive inference, since conformal prediction provides finite sample coverage guarantees for black box models and supports decision-making under dataset shifts.^16,17^ The remainder of the article details the evidence and curation logic, the modelling and interpretation workflow, and a decision-oriented presentation of how these tools can strengthen cellulose SPE planning and reporting across matrices.

## 2 Methodology

### 2.1 Data sources, screening, and eligibility

The evidence base was assembled through structured searches of Scopus, Web of Science Core Collection, PubMed, Science Direct, and Google Scholar, restricted to peer-reviewed journal articles in English published from 2015 to 2025 and complemented by backward and forward citation chasing. Screening followed PRISMA 2020, including duplicate removal, title and abstract screening, full text assessment against predefined criteria, and transparent flow reporting, and the platform-specific query templates are provided in Table S1.^9^ ROSES guidance was adopted to retain environmental evidence transparency in reporting decisions that are often underspecified in analytical method literature.^18^ SPIDER was used in eligibility structuring to prioritise method-centric reports with explicit evaluation metrics, and query blocks were used to combine cellulose terms, solid-phase extraction workflow terms, and validation descriptors aligned with the data dictionary.^19,20^

Language restriction and exclusion of grey literature, including theses, technical reports, and industrial protocols, were retained for traceability and permanent identifiers while recognising the risks of language bias and selective publication of positive outcomes.^21,22^ Eligibility required aqueous matrices implementing cellulose-based solid-phase extraction formats (cartridge, magnetic, dispersive, in-syringe, or paper-based), quantifying pharmaceuticals and reporting at least one endpoint for the recovery in matrix, enrichment factor, limit of detection, or limit of quantification, with procedural detail sufficient to map pH, sorbent chemistry and mass, eluent identity and volume, and instrument class. Adsorption-only studies without an extraction workflow were excluded from quantitative extraction because the modelling target is end-to-end extraction performance, but such studies informed the mechanistic framing used in interpretation.^19^

Matrix chemistry descriptors, including conductivity, dissolved organic carbon, UV_254_ absorbance, ionic composition, and salinity, were extracted when reported but were not mandatory because of pervasive under-reporting, a structural limitation expected to reduce transportability when matrix effects drive ion suppression and recovery variability.^23–25^ Quality indicators were recorded when explicit but were not used for weighting because quality appraisal and its downstream use remain inconsistent, and duplicates were collapsed by canonical DOI to define unique study identities for grouped validation in Table 1.^9,26^

**Table 1:** Eligibility framework and operational definitions

Domain	Inclusion criteria	Exclusion criteria	Operational notes
Source type	Peer-reviewed journal articles, English, 2015 to 2025, DOI available	Theses, conference abstracts, preprints, non-english, technical reports, industrial protocols	Filters applied, full texts checked for methods and identifiers
Sample and setting, SPIDER S	Aqueous matrices, river, lake, seawater, wastewater, groundwater, tap, ultrapure	Non-aqueous media, soils without aqueous loading, adsorption-only without extraction	Matrix category standardised, matrix chemistry fields extracted when reported
Phenomenon of interest, SPIDER PI	Cellulose-based solid-phase extraction of pharmaceuticals	Non-cellulose sorbents, blends without cellulose dominance	Cartridge, magnetic, dispersive, in-syringe, paper-based
Design, SPIDER D	Experimental extraction with detail to map pH, sorbent chemistry and mass, eluent identity and volume, and instrument class	Narrative pieces without procedural detail	Mapping follows the data dictionary
Evaluation, SPIDER E	Reports recovery in matrix, enrichment factor, limit of detection, or limit of quantification	No quantitative validation endpoints	Endpoints harmonised for modelling
Research type, SPIDER R	Primary empirical studies	Reviews without extractable datasets	Reviews screened to locate primary studies

### 2.2 Variable dictionary and role schema

The variable dictionary specified a harmonised set of fields that mirror the determinants of solid-phase extraction performance reported in recent cellulose-based extraction research and broader method development reviews.^7^ Each record included identifiers for study, year, country, and a canonical digital object identifier so that grouped cross-validation by study could be enforced downstream without leakage.^19^ Method variables captured controllable choices at loading and elution, including the pH at load, nominal conductivity of the matrix where available, sorbent chemistry class, sorbent mass, extraction format, eluent identity class, eluent pH, and eluent volume, together with instrument class. This is because these dimensions consistently govern retention, desorption, and detection outcomes in environmental workflows.^19,27^ Outcome targets were defined to match validation tables that are standard in environmental and pharmaceutical analysis: recovery in matrix as the primary endpoint and matrix to ultrapure recovery ratio (where both were reported), enrichment factor as a measure of preconcentration, and sensitivity endpoints, including limit of detection (LOD) and limit of quantification (LOQ), on the original concentration scale.^27^ Definitions and units followed the conventions in contemporary validation literature for liquid chromatography tandem mass spectrometry, with the explicit separation of recovery and matrix effects to avoid the conflation of extraction efficiency and ionisation artefacts.^28^

Analyte level descriptors included dominant p*K*_a_ values and distribution coefficients at the loading pH when a study reported these values or referenced a calculation, since the ionisation state and hydrophobicity drive interaction strength with cellulose-based phases and co-determine attenuation or carryover in aqueous systems.^7,19^ The inclusion of the instrument class, HPLC (with ultraviolet detection or fluorescence detection) or LC-MS/MS, reflected known differences in selectivity and susceptibility to matrix effects that influence apparent recovery and the reporting of low limits of detection and quantification.^29^ The matrix category field mapped descriptive phrases to a controlled vocabulary that distinguishes source waters, including river, lake, seawater, wastewater, groundwater, tap, and ultrapure, to enable matrix effect hypothesis tests that respect study identity.^19^

Optional matrix covariates were defined to capture natural organic matter and related surrogates when available, including ultraviolet absorbance at 254 nm and specific ultraviolet absorbance. This is because aromatic dissolved organic matter and its proxies modulate adsorption, recovery, and downstream ionisation in water analysis.^30,31^ Where reported, these variables were recorded as continuous predictors to support sensitivity analyses, noting their established use as indicators of dissolved organic matter character and treatability.^30^ Numeric predictors were stored as continuous variables in native units, with derived fields computed by fixed rules, such as total loaded mass as spike concentration times sample volume divided by one thousand and loaded mass normalised by sorbent mass when that mass was reported. Categorical predictors used controlled levels to stabilise encoding across studies and preserve compatibility with one-hot encoding and imputation that are applied later within model families that require such preprocessing.

### 2.3 Outcome definitions and transformations

The primary outcomes followed validation practice in environmental liquid chromatography: the recovery in matrix expressed as a percentage of the analyte measured after the full procedure relative to the fortified amount, as shown in eqn (1). A comparative normalisation to control for ionisation and extraction bias used the matrix to ultrapure recovery ratio in eqn (2), which is standard when matched ultrapure spikes are available.^32^ Preconcentration was represented by the enrichment factor operationally as the ratio of loaded sample volume to the final volume prior to injection, as shown in eqn (3).^33^ Sensitivity endpoints were the limits of detection and quantification on the concentration scale, estimated in practice either by signal-to-noise rules or calibration-based variance models, as summarised in eqn (4). Where distributions were strongly right-skewed, log 10 transforms were applied internally to stabilise variance for the enrichment factor and detection and quantification limits, while reporting and figures remained on the original scale.^34^ Definitions were implemented consistently across studies to permit pooled modelling while preserving the study identity for grouped validation.1
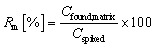
where *R*_m_ is the recovery in the matrix in percent, *C*_found,matrix_ is the measured concentration after extraction, and *C*_spiked_ is the fortified concentration.2
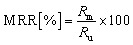
where MRR is the matrix to ultrapure recovery ratio, and *R*_u_ is the recovery in matched ultrapure water.3
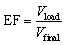
where EF is the enrichment factor, *V*_load_ is the sample volume loaded, and *V*_final_ is the final volume before injection.4
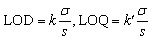
where *σ* is the residual standard deviation near the blank or low level, *s* is the calibration slope, *k* approximates three, and *k*′ approximates ten, consistent with contemporary estimation approaches.^32,34^

### 2.4 Preprocessing and feature engineering

Preprocessing followed a train-only policy to prevent information leakage from validation or test partitions, with all imputation and scaling parameters estimated on the current training indices and then applied to the corresponding validation split. Numeric predictors were first harmonised to the units defined in the variable dictionary and then inspected for implausible values that could arise from unit slips. Afterward, continuous missingness was handled by median imputation, stratified implicitly by the training data composition. Linear models received the standardisation of continuous inputs by centering and division by the training standard deviation, as shown in eqn (5), while tree-based learners consumed the raw, imputed scale to preserve the monotone structure.^35,36^

Categorical predictors were encoded by one-hot expansion with an explicit unseen category catch, and rare levels within a variable were consolidated using the controlled vocabulary defined during curation to stabilise parameter estimation across folds. Optional matrix covariates, such as the conductivity and surrogates of natural organic matter, for example, ultraviolet absorbance at 254 nm and specific ultraviolet absorbance (when reported) were treated as continuous predictors and passed through the same imputation and scaling logic as other numeric fields. Two derived fields were computed deterministically to capture loading intensity: total loaded mass from the product of spike level and sample volume and loaded mass normalised by sorbent mass when that mass was available, as given in eqn (6) and (7), respectively. These targets exhibit strong right skew, enrichment factor, and detection or quantification limits were modelled with internal log transforms solely within wrapped estimators to stabilise variance, while all reporting and figure generation remained on the original scale for interpretability.^36^5
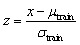
where *z* is the standardised value, *x* is the raw predictor, and *µ*_train_ and *σ*_train_ are the mean and standard deviation values estimated on the training data.6
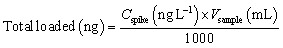
where *C*_spike_ is the spike concentration, and *V*_sample_ is the loaded sample volume.7
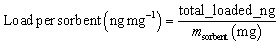
where *m*_sorbent_ is the sorbent mass entered for the extraction format.^35,36^

### 2.5 Validation design

Validation was structured to estimate out-of-study generalisation while preventing information leakage. A grouped outer cross-validation was implemented with study identity as the grouping unit so that all records from a given digital object identifier remained within the same fold, a design recommended for data with a hierarchical structure to avoid overly optimistic error estimates.^37^ Hyperparameter selection and model choice were confined to an inner cross-validation loop nested within each outer training split, providing an approximately unbiased assessment of generalisation error and mitigating selection-induced bias in performance estimates.^38^

All preprocessing, including imputation, scaling for linear estimators, and categorical encoding, was fitted exclusively on the current training indices and applied to the corresponding validation partition using pipeline encapsulation to eliminate leakage pathways.^39^ Model skill was summarised on outer folds using *R*^2^ and root mean square error (RMSE) as primary metrics, with mean absolute error (MAE) and rank-based association retained for sensitivity analyses. The same metric set was used for inner loop selection to maintain coherence between tuning and evaluation.^40^ Outer fold predictions and residuals were persisted to support two downstream diagnostics: calibration assessment *via* reliability curves that compare observed and predicted magnitudes and split conformal prediction to construct distribution-free prediction intervals at prespecified coverage levels.^41,42^ For targets modelled with internal monotone transforms to stabilise variance, back transformation was applied before scoring and calibration, so that all reported metrics and intervals reflect the original analytical scale relevant to method validation and planning.^41^

### 2.6 Model families and hyperparameter grids

Three complementary estimator families were prespecified to capture linear trends with embedded regularisation, nonlinear interactions with bagging, and boosted ensembles for flexible interaction depth. ElasticNet (EN) served as the linear baseline because the mixed L1 and L2 penalty stabilises coefficient estimates under collinearity and yields sparse, interpretable trends in chromatographic chemometrics.^43^ Random forest regressor (RF) was selected to model nonlinear relations and higher-order interactions that arise from simultaneous choices of pH at load, sorbent mass, eluent identity and volume, and matrix category in water analytics, with demonstrated robustness in environmental chemistry applications.^44^ Gradient boosted trees were represented by XGBoost to exploit additive tree ensembles with shrinkage, subsampling, and column sampling, offering a high performance for tabular experimental data at a modest computational cost.^45^

Hyperparameter grids were designed to span conservative to moderately complex settings while avoiding pathological regimes that promote overfitting on grouped folds. For ElasticNet, the grid traversed the regularisation strength and mixing ratio over logarithmic scales that are widely used in spectroscopic and chromatographic modelling.^43,45^ For random forest, the grid balanced forest size against depth and node size to trade bias and variance in structured environmental predictors.^44^ For XGBoost (XGB), the grid explored estimators, learning rate, maximum depth, subsampling, and column sampling, and both L1 and L2 regularisation to map the stable basins of skill seen in recent benchmarking.^45^ The final grids and preprocessing expectations per family are summarised in Table 2, which documents parameter names, ranges, and notes for reproducibility and the exact recreation of the search space in other software. To situate these choices within current optimisation practice, the table also indicates parameters that are most often refined by Bayesian or evolutionary search in follow-up sensitivity studies when computational budgets permit.^46^

**Table 2:** Model families, preprocessing expectations, and hyperparameter grids for the grouped cross-validation workflow

Family	Rationale and preprocessing	Primary hyperparameters and ranges
ElasticNet	Linear baseline with embedded variable selection, numeric features standardised within fold, categorical one-hot encoded with unknown catch	Alpha in [0.001, 0.01, 0.1, 1.0, 10.0], L1 ratio in [0.1, 0.3, 0.5, 0.7, 0.9], fitintercept = true
Random forest regressor	Nonlinear interactions and robust averaging, numeric features used on native imputed scale, categorical one-hot	Nestimators in [300, 600, 900], maxdepth in [none, 10, 20, 40], minsamplessplit in [2, 5, 10], minsamplesleaf in [1, 2, 4], maxfeatures in [sqrt, log 2, none]
XGBoost regressor	Additive boosted trees with shrinkage and subsampling, numeric on native imputed scale, categorical one-hot	Nestimators in [400, 800, 1200], learningrate in [0.01, 0.05, 0.1], maxdepth in [3, 5, 7], subsample in [0.6, 0.8, 1.0], colsamplebytree in [0.6, 0.8, 1.0], reg_alpha in [0.0, 0.1, 1.0], reg. lambda in [0.1, 1.0, 5.0]

### 2.7 Study metrics

Model skill was quantified on outer validation folds using complementary criteria that balance explained variance, absolute error magnitude, relative error sensitivity, and rank concordance. *R*^2^ measured the fraction of variance in the outcome explained by predictions and is scale-free, which aids comparison across targets reported in shared units (see eqn (8)). As expressed in eqn (9), the RMSE emphasised larger deviations and preserved the original analytical scale used for planning. The MAE provided a robust central tendency of error magnitudes (eqn (10)). The mean absolute percentage error (MAPE) summarises proportional error while protecting against division by near-zero values through a small constant (eqn (11)). Rank association was assessed with the Spearman correlation (*ρ*) to capture monotonic agreement between predicted and observed values independent of marginal distributions (eqn (12)).^47,48^8
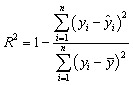
where *y*_*i*_ are observations, *ŷ*_*i*_ are predictions, and *ȳ* is the sample mean.9
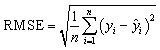
10
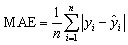
11
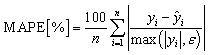
where *ε* is a small constant to avoid division by zero.

12 *ρ*_s_ = corr(rank(*y*), rank(*ŷ*))

where *ρ* is the Spearman rank correlation coefficient.

### 2.8 Matrix effect testing

Matrix-related bias was interrogated at two complementary levels: a distribution-free comparison of residuals across matrix categories and a hierarchical model that estimated adjusted contrasts while accounting for between-study heterogeneity. Nonparametric testing used the Kruskal–Wallis procedure on outer fold residuals grouped by matrix category, followed by Dunn-type pairwise comparisons with family-wise error control *via* Holm adjustment, a combination recommended for unequal variances and non-normal error structures common in environmental analytics.^49^ Effect sizes were summarised as median differences with percentile bootstrap confidence intervals to provide interpretable magnitude estimates alongside significance decisions, consistent with recent guidance emphasising interval estimation for method evaluation.^27,49^

To isolate systematic matrix shifts from study-specific baselines, a linear mixed effects model was fitted with the matrix as a fixed effect and random intercepts by study identifier, enabling the estimation of marginal means by matrix while preserving the grouping inherent to multi-analyte validation tables.^50^ Estimated marginal means and their pairwise contrasts, adjusted for multiplicity with Holm's method, were extracted to provide model-based summaries that are robust to unbalanced designs typical of published extraction studies.^49^ Where conductivity or optical surrogates of natural organic matter, such as ultraviolet absorbance at 254 nm or specific ultraviolet absorbance, were available, these variables were introduced as covariates to test attenuation or enhancement hypotheses that are mechanistically linked to ion suppression and recovery variability in LC-MS workflows.^51^ Plots from published extraction studies fit *versus* observed residual distributions by matrix and displayed contrast estimates with confidence intervals to guide interpretation without relying on distributional assumptions beyond those required by the mixed model.^50^

### 2.9 Feature attribution

Feature attribution proceeded along two complementary tracks to balance explanatory depth with robustness under correlated design variables. For tree-based ensembles, Shapley additive explanations were computed using the TreeSHAP algorithm to obtain locally faithful attributions that aggregate to coherent global importance profiles for sorbent and method variables.^52^ Local contributions were summarised to median absolute Shapley values at the feature level and, where appropriate, to grouped contributions for domain constructs, such as matrix class, eluent family, and sorbent chemistry, to respect the scientific hierarchy of the workflow.^52,53^

For all estimator families, model agnostic permutation-based importance was applied on the outer validation folds by permuting a feature within the fold and recording the change in the selected loss, with grouping by study to preserve correlation structure and to prevent information leakage across studies.^53^ To mitigate the known sensitivity of permutation measures under multicollinearity, importance was reported alongside model class reliance style bounds and, where required, group-wise permutation was used to probe the reliance on the blocks of related variables, such as pH and conductivity or eluent identity and eluent volume.^54^ All attribution calculations were nested inside the cross-validation so that preprocessing, model fitting, and importance estimation were learned on training indices and evaluated on held-out data only, which aligns with current recommendations for leakage-free interpretability in supervised chemometric settings.^53^

### 2.10 Calibration, conformal prediction, and reproducibility assets

Calibration assessed agreement between the predicted magnitudes and observed outcomes by reliability analysis that bins predictions within each outer validation fold and compares the mean predicted and mean observed values on the original analytical scale, with any monotone transforms inverted before scoring.^55^ Distribution-free prediction intervals were obtained using split conformal methods that calibrate interval width on residuals from the training partition, with jackknife^+^ used as a sensitivity check in settings with few studies or heterogeneous design and conformalised quartile regression applied where heteroscedastic noise was suspected.^56,57^ Coverage diagnostics reported empirical inclusion rates with binomial confidence intervals and conditional checks across matrix categories.^55,57^

Reproducibility assets were engineered to satisfy findable, accessible, interoperable, and reusable expectations by versioning the curated analysis matrix, role schema, fitted preprocessing and estimator pipelines, and all evaluation tables and figure specification files as comma-separated values suitable for direct import to external plotting software.^58,59^ Determinism was promoted through fixed random seeds, a pinned requirements file, checksums for inputs and outputs, and path-stable scripts that resolve project roots and write to audited subfolders.^58^ Documentation comprised a run manifest describing inputs, parameter grids, validation folds, and file checksums to permit independent verification and lower the barrier to external reuse in related environmental analytics.^59^ The computational environment comprised Python version 3.11.0 in a dedicated virtual environment within Visual Studio Code on Windows 10 64 bit, using NumPy, pandas, SciPy, and scikit learn for data processing, pipelines, and grouped validation, XGBoost for boosted trees when available, stats models for mixed effects when available with a validated fixed effects fallback, SHAP for tree-based explainability when feasible, permutation importance for all estimators, and Matplotlib for verification plots. Journal-grade figures were produced in Origin 2024 by importing the saved figure specification files to ensure consistent axes, labels, and colour decisions.^52,58^

## 3 Results and discussion

### 3.1 Evidence-based overview and data completeness

**Fig. 1:**
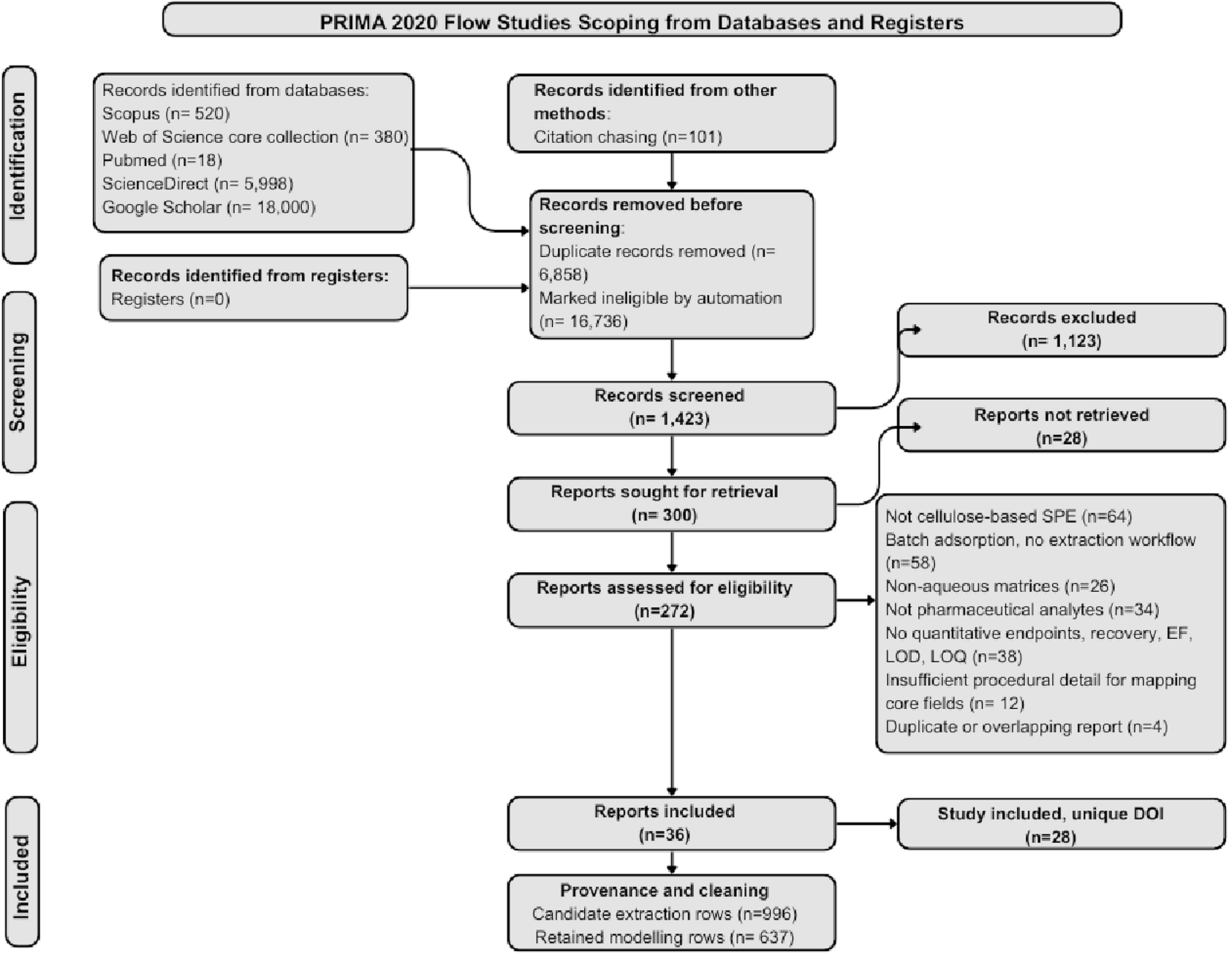
PRISMA 2020 study identification, screening, and inclusion flow for cellulose-based solid-phase extraction studies on pharmaceuticals in aqueous matrices, with a cleaning tail linking included reports to the retained modelling dataset.

The consolidated analysis matrix contains 637 experimental rows and 29 variables mapped to a controlled dictionary, representing 28 unique digital object identifiers that survived screening and harmonisation from an initial 996 candidate rows drawn across 36 studies, as detailed in Table S2. The detailed PRISMA flow for the search and study inclusion and exclusion process, with reasons, is illustrated in Fig. 1. The role schema defines five supervised endpoints: recovery in matrix, matrix to ultrapure recovery ratio, enrichment factor, and the sensitivity pair LOD and LOQ, alongside 25 candidate predictors spanning matrix identity, sample conditioning, sorbent formulation, elution chemistry, and instrumental platform, which provides a balanced basis for both prediction and inference without inflating the feature to sample ratio. Completeness is heterogeneous across families. Sample volume and enrichment factor are reported for about 630 rows. The sensitivity pair appears for about 520 LOD rows and 458 LOQ rows, while eluent pH is disclosed in only a few tens of cases, which is consistent with recent method validation surveys that note the incomplete reporting of solvent additives and pH schedules in water methods.^60^

Matrix descriptors that control ion pairing and signal suppression are sparse. Conductivity is present in a minority of studies, and natural organic matter surrogates, such as specific ultraviolet absorbance at 254 nm, are essentially absent. This aligns with wider observations that the routine characterisation of natural organic matter is underreported in environmental workflows and that this gap can limit the mechanistic attribution of matrix effects.^61^ These omissions matter because matrix-dependent ionisation and co-extracted organic matter are primary drivers of signal suppression or enhancement in liquid chromatography tandem mass spectrometry and should be considered when interpreting cross matrix comparability.^4,61^

The evidence base spans multiple extraction formats and cellulose chemistries, with cartridge, magnetic, and paper-based implementations represented together with unmodified, carboxylated, and composite cellulose scaffolds. This ensures that downstream models see realistic diversity in retention and desorption mechanisms rather than single laboratory optimisations. This breadth is essential because predictive skill and interval coverage should be established on data that respects study identity and analytical context, a practice emphasised in contemporary validation guidance for environmental analysis.^62^ The cleaned matrix also retains explicit identifiers at the level of study and analyte, which enables fold assignment by digital object identifier (DOI) and preserves the out-of-study evaluation in later sections without leakage between the training and validation strata, an approach that aligns with current expectations for transparent model evaluation in analytical chemometrics.^63^ The missingness audit detailed in Table S3 records columns with more than 10% gaps by family and annotates likely consequences for model covariate selection and interpretation of matrix contrasts. In combination with the distributions and endpoint coverage in the dataset structure consolidation, these diagnostics establish the scope and limits of the curated evidence, frame the interpretation of matrix effects in Section 3.3, and justify the grouped cross-validation and mixed effects strategies that follow.

### 3.2 Model performance and prediction fidelity

**Fig. 2:**
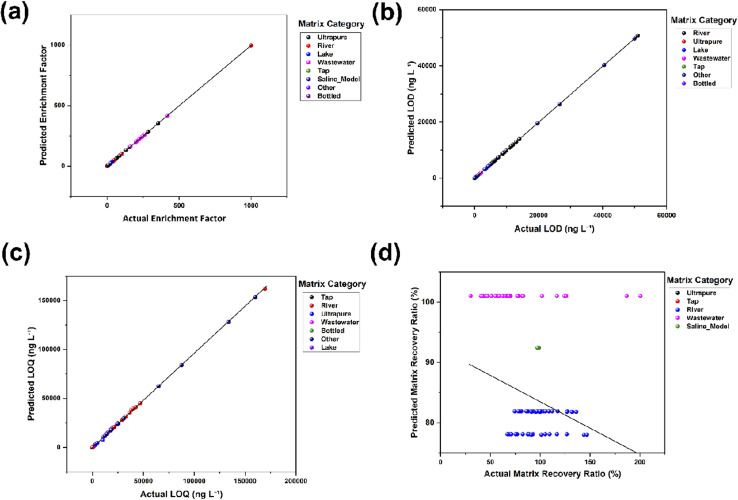
Predicted *versus* observed for each target: (a) enrichment factor, (b) LOD, (c) LOQ, and (d) matrix recovery ratio, with points coloured by matrix category.

Model selection at the target level identified ElasticNet as the dominant predictor for sensitivity endpoints and enrichment, while random forest emerged as the least poor alternative for the two recovery targets, as summarised in Table 3. Across the combined outer validation partitions shown in Fig. 2, ElasticNet achieved near-perfect alignment for the enrichment factor with a mean *R*^2^ of 0.99999 (Fig. 2a) and a root mean square error (RMSE) of 0.296 units, based on 630 out-of-study predictions pooled across folds, with a Spearman correlation of 0.987, indicating monotonic fidelity in addition to low dispersion. In the same design, ElasticNet reached *R*^2^ values of 0.9999 for the LOD (Fig. 2b) and 0.9991 for the LOQ (Fig. 2c), with RMSE values of 23.9 and 502.4 ng L^−1^, respectively, supported by 520 and 458 outer fold predictions. All three figure parts illustrate tight adherence to the identity line, with only minor spread at the extremes, consistent with stable calibration across folds.

Predictive performance summary by target. Mean and standard deviation across outer folds for *R*^2^, RMSE, MAE, MAPE, and Spearman rank correlation, and the direct model leaderboard overview of the different targets

**Table:** 

Target	Champion model	Selected hyperparameters	*R* ^2^ (mean ± SD)	RMSE (mean ± SD)	MAE (mean ± SD)	MAPE (mean ± SD)	Spearman (*ρ*) (mean ± SD)
Recovery matrix (%)	Random forest regressor	N estimators = 300 (300–900); max depth = 10 (NA/10); max features = sqrt (sqrt/log 2); min samplessplit = 5 (2–10); min samplesleaf = 4 (1–4)	−0.193 ± 0.263	18.92 ± 9.60	13.86 ± 6.57	41.98 ± 46.06	0.072 ± 0.226
Matrix recovery ratio	Random forest regressor	N estimators = 300 (300–900); max depth = 10 (NA/10); max features = log 2; min samplessplit = 2 (2–10); min samplesleaf = 2 (1–4)	−24.422 ± 41.325	26.25 ± 20.77	22.29 ± 18.45	31.76 ± 35.54	−0.078
Enrichment factor	ElasticNet	Alpha = 0.001; L1ratio = 0.9 (0.5–0.9); fit intercept = true	0.99999 ± 0.000	0.30 ± 0.34	0.11 ± 0.09	0.10 ± 0.04	0.987 ± 0.029
LOD matrix (ng L^−1^)	ElasticNet	Alpha = 0.001; L1ratio = 0.9 (0.7–0.9); fit intercept = true	0.99985 ± 0.000	23.87 ± 23.90	9.19 ± 9.35	0.63 ± 0.89	1.000 ± 0.000
LOQ matrix (ng L^−1^)	ElasticNet	Alpha = 0.001 (0.001–0.01); L1 ratio = 0.9; fit intercept = true	0.99914 ± 0.001	502.39 ± 945.13	246.06 ± 503.99	2.08 ± 1.72	0.994 ± 0.005

**Table:** 

Leaderboard overview						
Target	Model	*R* ^2^	RMSE	MAE	MAPE	Spearman r
Recovery matrix (%)	Random forest regressor	−0.19302	18.92351	13.85523	41.97597	0.072217
Recovery matrix (%)	XGB regressor	−0.55384	19.78893	14.17685	44.71336	0.13969
Recovery matrix (%)	ElasticNet	−0.63659	20.14639	15.11677	43.82396	0.196057
Matrix recovery ratio	Random forest regressor	−24.4217	26.24956	22.29039	31.7566	−0.0782
Matrix recovery ratio	XGB regressor	−184.928	26.38633	22.08044	28.8264	0.11973
Matrix recovery ratio	ElasticNet	−88969.1	136.5824	132.5592	143.4689	
Enrichment factor	ElasticNet	0.999993	0.296366	0.107931	0.102189	0.987051
Enrichment factor	XGB regressor	0.692411	50.69821	15.84766	11.25536	0.936858
Enrichment factor	Random forest regressor	0.616211	62.61097	21.91674	19.6515	0.889016
LOD matrix (ng L^−1^)	ElasticNet	0.999851	23.86571	9.187872	0.627696	0.999825
LOD matrix (ng L^−1^)	Random forest regressor	0.865071	1372.57	455.9437	32.93353	0.960757
LOD matrix (ng L^−1^)	XGB regressor	0.84593	2142.793	668.2255	24.19606	0.981715
LOQ matrix (ng L^−1^)	ElasticNet	0.999137	502.3898	246.0563	2.083099	0.993771
LOQ matrix (ng L^−1^)	Random forest regressor	0.850956	7646.139	2768.322	23.12209	0.973439
LOQ matrix (ng L^−1^)	XGB regressor	0.825911	7659.36	2881.406	23.003	0.966004

In contrast, the two recovery outcomes proved challenging. For absolute recovery in the matrix, random forest topped the leaderboard with a negative *R*^2^ of about −0.19 and an RMSE of 19 %pt. However, ElasticNet delivered a slightly better pooled scatter in the project-specific predictions yet still returned a negative *R*^2^ of −0.079 and an RMSE of 21.5 %pt over 513 validation rows. The matrix-over-ultrapure recovery ratio exhibited even lower predictive skill, with random forest boosting a mean *R*^2^ near −24 (Fig. 2d) and an RMSE of around 26 %pt across folds, and all the models showed Spearman coefficients close to zero. A negative *R*^2^ in this out-of-study setting indicates that recovery predictions do not outperform a mean-only baseline, which is consistent with recovery being dominated by unreported matrix constituents and protocol idiosyncrasies that inflate irreducible error. Current reviews emphasise that matrix effects in liquid chromatography tandem mass spectrometry arise from co-extracted salts, natural organic matter, and surfactants that alter ionisation efficiency and can invert apparent trends across contexts, which degrades transportability without explicit matrix covariates or matrix-matched calibration.^64^

The divergent behaviour between sensitivity and enrichment on one side and recovery on the other has practical and mechanistic implications. The sensitivity endpoints and enrichment are largely governed by definitional relationships and near-deterministic manipulations of load and eluent volumes, pH transforms, and log scale mappings, which favour linear models with explicit regularisation and reduce variance under grouped cross-validation. The result is a narrow, high-performing basin where simple models dominate, consistent with the tight predicted *versus* observed clouds and the compressed residual distributions for these targets. Recovery, by contrast, integrates sorption kinetics, competition with dissolved organic carbon, ionic strength, and co-extracted colloids, with many studies not reporting conductivity, DOC proxies, or UV_254_ absorbance. In such settings, group-aware validation is essential to prevent information leakage across studies. However, even a strict leave-group-out evaluation cannot compensate for missing causal drivers, resulting in the negative *R*^2^ landscape observed here.^6,64^

Overall, the evidence indicates that enrichment and detection limits can be forecast with high fidelity from the curated controllable variables, while recovery outcomes remain only weakly predictable under the present reporting practices.^64^ This divergence supports a dual strategy in subsequent sections, namely to continue with ElasticNet for sensitivity and enrichment where confidence is warranted and then pivot to error mapping, non-parametric contrasts, and mixed effects confirmation for recovery. This will quantify matrix-linked dispersion and identify robust operating windows rather than point predictions.

### 3.3 Matrix-specific performance and residual structure

**Fig. 3:**
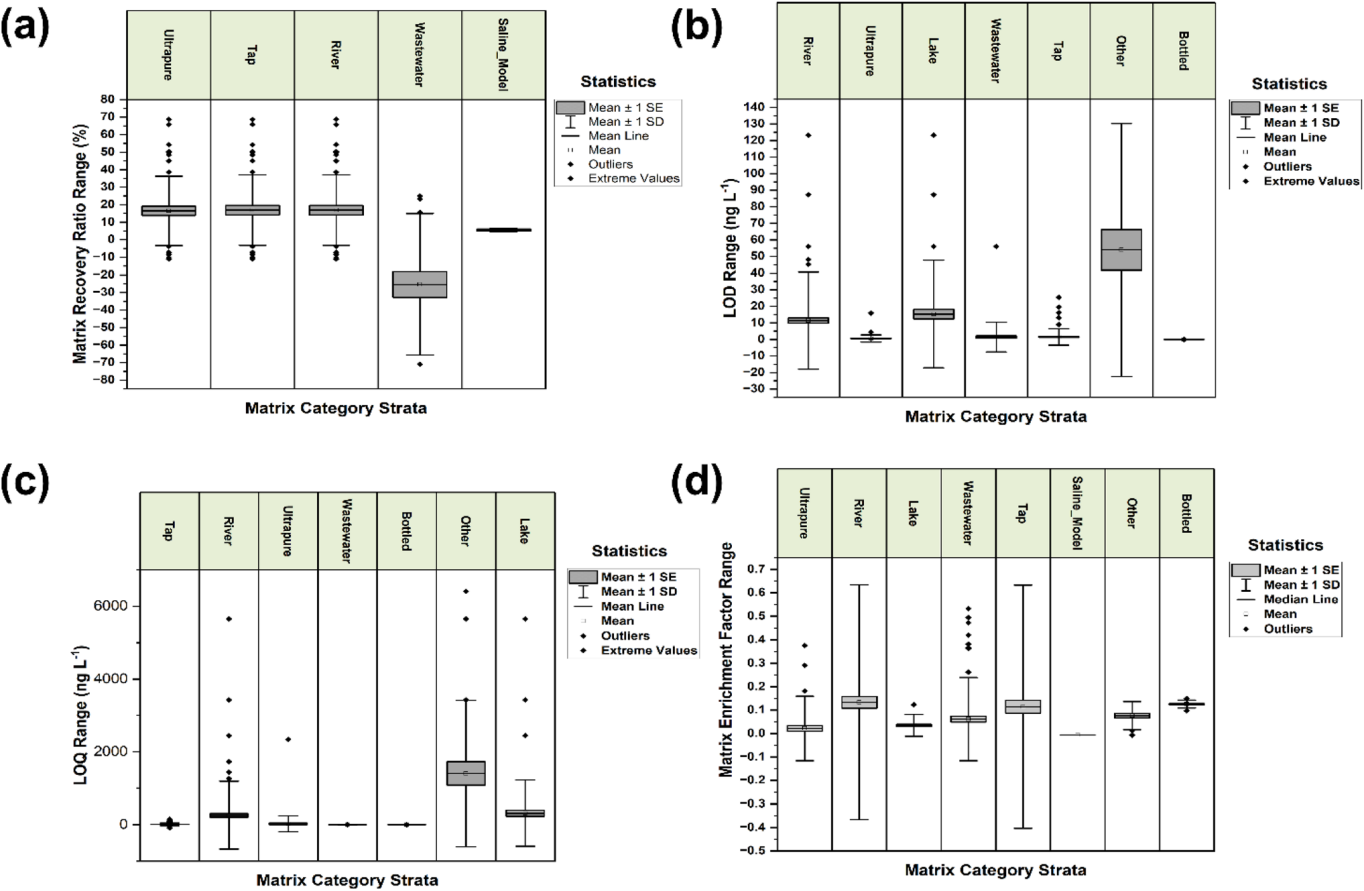
Box plot of residuals by matrix showing the distribution of outer fold residuals stratified by matrix category for each target: (a) matrix recovery ratio, (b) LOD, (c) LOQ, and (d) enrichment factor.

Across matrices, the residual structure reflected marked differences in both central tendency and dispersion that align with the chemical heterogeneity of natural waters and the comparative simplicity of laboratory waters. For the recovery in matrix, the champion model exhibited the largest residual spread in wastewater and river categories, with wastewater showing an outer fold root mean square error (RMSE) of approximately 31% and a MAE near 22% over 147 instances, whereas river presented a RMSE near 17% and a MAE near 13% over 139 instances. Ultrapure water displayed the tightest behaviour with a RMSE and MAE around 12% and 9% over 56 instances, respectively, indicating greater fidelity where matrix components are minimal (see Table S4). The bootstrap summaries by matrix corroborated these trends and further indicated lake water as an intermediate case, with a RMSE of about 16.8% and a MAE near 13.4% across 48 observations, consistent with moderate but non-negligible matrix complexity, as detailed in Table S4. Distributional views of the outer fold residuals showed visibly wider boxes and longer upper whiskers for wastewater and river relative to ultrapure and tap waters, underscoring asymmetry and occasional high positive deviations characteristic of under-prediction at an elevated matrix burden (see detailed illustration in Fig. 3).

Non-parametric contrasts confirmed statistically meaningful differences between key matrix pairs for recovery in the matrix. The omnibus Kruskal procedure, followed by Dunn comparisons with Holm adjustment, yielded large effect sizes for lake *versus* wastewater and river *versus* wastewater, with Cliff delta magnitudes around 0.70 and 0.49, respectively, at adjusted probabilities below 0.01, indicating systematically higher central recovery behaviour in lake and river relative to wastewater within the curated evidence set. This is all well detailed in Table S5. River *versus* ultrapure also showed a moderate to large effect with an adjusted probability close to 0.01, consistent with the presence of residual bias in natural flowing waters not evident under laboratory conditions.

For the matrix recovery ratio target illustrated in Fig. 3a, performance degraded relative to absolute recovery because of the restricted subset requiring paired ultrapure measurements. Wastewater showed a negative coefficient of determination with a RMSE of about 47 %pt over 56 rows, while the ultrapure subset, though smaller, retained wide residuals around 26 %pt. This indicated that the available covariates did not fully capture ratio variability and that the signal-to-noise trade-off penalised predictive fidelity in this reduced stratum (detailed in Table S4). Nevertheless, the rank-based contrasts still resolved substantial differences, with river *versus* wastewater and tap *versus* wastewater exhibiting Cliff delta values near 0.72 and 0.65 at adjusted probabilities under 0.01, implying consistently larger ratios outside wastewater and pointing to stronger matrix suppression characteristics under wastewater conditions.

The sensitivity endpoints mirrored this matrix ordering. For the limit of detection (Fig. 3b), river displayed a RMSE of around 31 ng L^−1^ across 80 rows, while wastewater remained near 0.15 ng L^−1^ across 82 rows, and bottled water was essentially negligible near 0.01 ng L^−1^ across 28 rows. This indicated strong stability where ionic and organic backgrounds are minimal and a broad, right-skewed error field in natural rivers. The limit of quantification (illustrated in Fig. 3c), which is similar to that of the enrichment factor (Fig. 3d) showed the largest dispersion in river, with a RMSE near 1220 ng L^−1^ across 82 rows, compared with 0.10 ng L^−1^ in wastewater and about 3 ng L^−1^ in bottled water, highlighting a pronounced tail of under-prediction events in flowing natural waters that concentrate variance at the upper end (Table S4). Corresponding residual distributions revealed median residuals near 2 ng per litre in river with wide interquartile spans exceeding 40 ng per litre, contrasted with medians near zero and narrow interquartile ranges in wastewater and bottled water, further emphasising the impact of matrix heterogeneity on sensitivity calibration. Conductivity annotations were sparse, limiting a fully continuous attribution of ionic strength effects; nevertheless, the matrix ordering and large effect contrasts observed for wastewater against river, lake, and tap converge on a practical interpretation that high and variable co-extracted constituents in environmental waters drive both the residual bias and error width, whereas simplified matrices approach nominal behaviour.^65,66^

### 3.4 Mixed effects confirmation and between-study heterogeneity

**Fig. 4:**
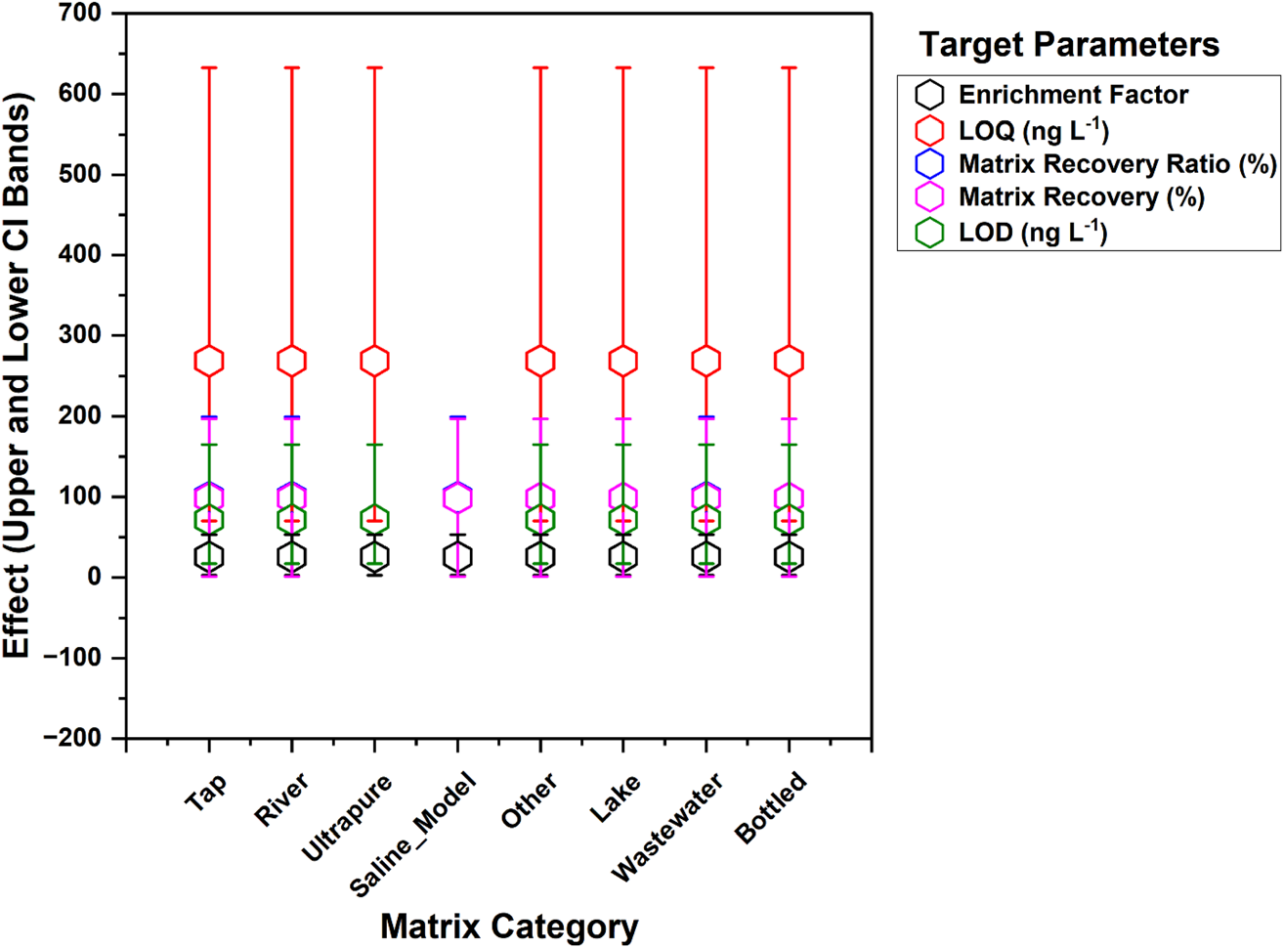
Matrix fixed effects forest. Fixed effect estimates with 95% confidence intervals (CI) relative to a reference matrix.

Hierarchical modelling with study encoded as a random intercept confirmed that most between-study variability resides at the study level rather than the matrix level for the principal recovery endpoints, while the sensitivity endpoints retained non-trivial study-specific dispersion. The mixed effects summaries show the random intercept variance for the recovery in matrix and matrix-over-ultrapure ratio estimated near zero. This indicates limited unexplained heterogeneity at the study level for these endpoints after conditioning on the fixed set of method and analyte covariates, as illustrated in Fig. 4. Conceptually, such patterns are consistent with a model where systematic differences in recovery are already captured by controllable factors and analyte descriptors, so the residual study effect is small.^60,67,68^ The marginal and conditional *R*^2^ interpretation for mixed models follows a study by Nakagawa and coauthors.^69^

Estimated marginal means for the matrix categories were nearly identical within each endpoint, and the confidence intervals overlapped across matrices. This implies that once the study and covariates are accounted for, the average predicted recovery and sensitivity do not differ materially by matrix category. For the recovery in matrix, all matrix categories concentrate near an adjusted mean of about 98%. For the ratio of the matrix to ultrapure recovery, the adjusted means cluster close to 100%. For the enrichment factor, the adjusted means cluster near 25. For the limits of detection and quantification, the adjusted means concentrate near 72 and 269 ng L^−1^, respectively. The use of the adjusted means provides a principled way to compare matrix categories on a common covariate baseline and is standard in mixed model reporting.^70^

Transportability across studies, evaluated with leave-one-study-out validation, confirms that out-of-study prediction is non-trivial for all endpoints, with negative median explained variance across held-out DOIs and broad interquartile ranges in error (see Table 4). These patterns align with methodological evidence that grouped cross-validation can reveal genuine between-study heterogeneity and that model skill often degrades under distribution shift when protocols, instruments, and reporting practices differ across sites.^71,72^ In the recovery matrix (%), 17 DOIs qualified (median held-out *n* = 24), yielding a median *R*^2^ of −0.717 with an IQR of −2.061 to −0.101 and a median RMSE of 7.737 %pt (5.064–9.508). The matrix recovery ratio endpoint was evaluable in only two DOIs (median held-out *n* = 42) and showed extreme instability, with a median *R*^2^ of −5.18 × 10^4^ (IQR of −7.76 × 10^4^ to −2.59 × 10^4^) and a median RMSE of 1.12 × 10^3^ (564–1675), underscoring how sparse matched ultrapure recovery and ratio amplification can magnify study-specific idiosyncrasies.

**Table 4:** Leave-one-study-out performance tabulation showing R^2^ and RMSE across held-out DOIs with median and interquartile range (*Q*_1_–*Q*_3_)

Target	LOIO approach used	Held-out DOIs (*n*)	Median test *n* per DOI	*R* ^2^ median (*Q*_1_–*Q*_3_)	RMSE median (*Q*_1_–*Q*_3_)
Recovery matrix (%)	Mixed-effects LOIO	17	24	−0.717 (−2.061 to −0.101)	7.737(5.064–9.508)
Matrix recovery ratio	Mixed-effects LOIO	2	42	−51,753 (−77 629 to −25 877)	1120 (563.8–1675)
Enrichment factor	Mixed-effects LOIO	25	19	−0.328 (−11.00–0.640)	1.295 (0.6343–1.726)
LOD matrix (ng L^−1^)	Mixed-effects LOIO	19	21	−5.738 (−14.18–0.345)	1.698 (1.465–3.483)
LOQ matrix (ng L^−1^)	Mixed-effects LOIO	17	21	−0.818 (−5.128–0.868)	1.018 (0.6656–3.086)

Consistent with this theme, the enrichment factor, LOD matrix (ng L^−1^), and LOQ matrix (ng L^−1^) exhibited negative median *R*^2^ values of −0.328, −5.738, and −0.818, respectively, despite upper-quartile values above zero for some targets. This indicates that a subset of studies remains more predictable under the current covariate set (detailed in Table 4). The corresponding median RMSE values were 1.295 (0.634–1.726), 1.698 (1.465–3.483), and 1.018 (0.666–3.086) in the modelling scale used for cross-study validation. Taken together, these results reinforce that fixed matrix categories and reported method descriptors capture only part of the variance driving inter-study performance, with residual differences likely absorbed by the random-intercept and other study-level components. Mixed-effects modelling remains appropriate for estimating adjusted contrasts while respecting within-study dependence in multi-study chemical datasets.^73^ From a method development perspective, the findings support the more routine reporting of matrix chemistry proxies (for example, conductivity, UV_254_, and major-ion profiles) and harmonised recovery definitions to improve future evidence synthesis and transportability.^72^

### 3.5 Drivers of performance and mechanistic interpretation

**Fig. 5:**
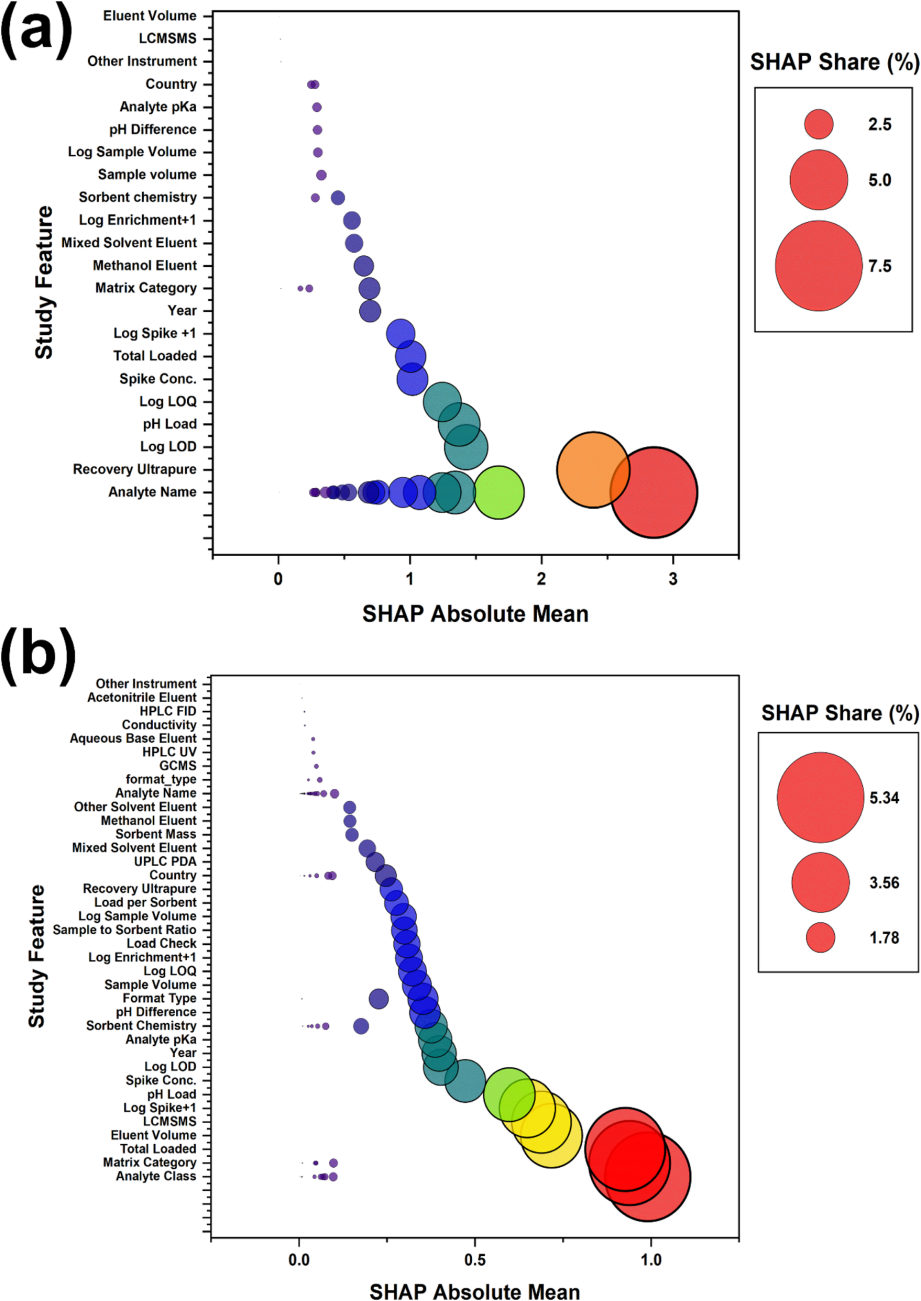
SHAP summary for the primary target: (a) matrix recovery and (b) matrix recovery ratio.

Permutation importance and Shapley attribution together outline a consistent hierarchy of predictors, with chemical identity, matrix descriptors, and study descriptors explaining most of the variance in the recovery endpoints, while operational settings provide secondary but useful control over error magnitude.^74^ For the recovery in the matrix presented graphically in Fig. 5a, the random forest Shapley summaries assign about 7.4% of the total attribution to the analyte class and about 6.2% to the matrix category, with total loaded mass, sorbent chemistry, and eluent volume contributing roughly 5.0, 4.1, and 3.9%, respectively, and study-level identifiers capturing an additional substantial share. This allocation is consistent with sorption frameworks in which molecular functionality and the ionisation state govern interactions with charged and aromatic regions on cellulose-based phases, while eluent volume modulates desorption completeness and apparent recovery.^7^ The prominent role of the matrix category should be interpreted as a coarse proxy for the salinity and co-extracted organic matter that influence extraction equilibria and ionisation efficiency in liquid chromatography-tandem mass spectrometry, rather than as a fully specified description of matrix chemistry.^75^ This framing directly aligns the attribution patterns with a mechanism-first explanation because the model is effectively learning how a limited set of reported variables stands in for stronger but missing drivers, including conductivity-linked ionic strength, dissolved organic carbon, and UV_254_ proxies, which are frequently absent from method validation sections.^6,75^

Permutation importance at the parent feature level reinforces this perspective. For the recovery in matrix, permuting the study identifier and DOI reduces the mean outer fold *R*^2^ by approximately 0.056 and 0.045, respectively. However, the analyte class and matrix category cause drops of around 0.034 and 0.026, respectively, and eluent volume, instrument class, and total loaded mass each reduce the *R*^2^ by about 0.02. This performance average is detailed in Table 5. The prominence of study-level identifiers indicates that part of the predictive skill reflects latent protocol, laboratory practice, and reporting structure that differ systematically between studies, rather than transferable physicochemical causality.^72^ In this setting, study and DOI are treated as stratification markers rather than tunable method variables, and their influence is presented as a diagnostic signal of inter-study heterogeneity, not as a recommendation for protocol optimisation.^68^ This interpretation is also consistent with the observed instability of recovery models under strict grouped validation because strong study signatures and weak matrix chemistry reporting jointly constrain transportability even when leakage is controlled.^6,64,72^

**Table 5:** Average performance degradation when permuting each feature, aggregated over outer folds for champion models

Target	Champion model	Feature	Importance (mean ± SD)
Recovery matrix (%)	Random forest regressor	Study ID	0.0557 ± 0.004
Recovery matrix (%)	Random forest regressor	DOI	0.0447 ± 0.00293
Recovery matrix (%)	Random forest regressor	Analyte class	0.034 ± 0.00568
Recovery matrix (%)	Random forest regressor	Matrix category	0.0262 ± 0.00442
Recovery matrix (%)	Random forest regressor	Eluent volume (mL)	0.0233 ± 0.0027
Recovery matrix (%)	Random forest regressor	Instrument	0.0222 ± 0.00436
Recovery matrix (%)	Random forest regressor	Total loaded (ng)	0.0222 ± 0.00267
Recovery matrix (%)	Random forest regressor	Sorbent chemistry	0.0161 ± 0.00173
Recovery matrix (%)	Random forest regressor	Log spike +1	0.0159 ± 0.00168
Recovery matrix (%)	Random forest regressor	Analyte name	0.0145 ± 0.00109
Matrix recovery ratio	Random forest regressor	Analyte name	0.323 ± 0.0294
Matrix recovery ratio	Random forest regressor	Recovery ultrapure (%)	0.137 ± 0.0111
Matrix recovery ratio	Random forest regressor	Notes	0.122 ± 0.0148
Matrix recovery ratio	Random forest regressor	Analyte class	0.0591 ± 0.0101
Matrix recovery ratio	Random forest regressor	Log LOQ	0.0384 ± 0.00466
Matrix recovery ratio	Random forest regressor	Log LOD	0.0371 ± 0.0041
Matrix recovery ratio	Random forest regressor	Eluent identity class	0.0256 ± 0.00437
Matrix recovery ratio	Random forest regressor	Matrix category	0.0247 ± 0.0038
Matrix recovery ratio	Random forest regressor	pH load	0.0205 ± 0.00926
Matrix recovery ratio	Random forest regressor	Total loaded (ng)	0.0179 ± 0.00454
Enrichment factor	ElasticNet	Log enrichment +1	2 ± 0.0764
LOD matrix (ng L^−1^)	ElasticNet	Log LOD	1.93 ± 0.109
LOD matrix (ng L^−1^)	ElasticNet	Log LOQ	0.000692 ± 0.000117
LOQ matrix (ng L^−1^)	ElasticNet	Log LOQ	1.48 ± 0.0597
LOQ matrix (ng L^−1^)	ElasticNet	Log LOD	0.117 ± 0.0209
LOQ matrix (ng L^−1^)	ElasticNet	Load per sorbent (ng mg^−1^)	7.39 × 10^−5^ ± 0.000174
LOQ matrix (ng L^−1^)	ElasticNet	Year	4.83 × 10^−6^ ± 7.11 × 10^−7^
LOQ matrix (ng L^−1^)	ElasticNet	Load check (ng mg^−1^)	4.28 × 10^−6^ ± 2.51 × 10^−6^
LOQ matrix (ng L^−1^)	ElasticNet	Log spike +1	1.32 × 10^−6^ ± 3.87 × 10^−7^

The recovery ratio models shown in Fig. 5b, which contrast environmental matrices with ultrapure water, are even more strongly driven by intrinsic analyte properties. Shapley aggregation assigns about 30.6% of the contribution to the analyte name and about 7.7% to the analyte class, whereas baseline ultrapure recovery contributes about 6.4%, loading pH about 3.7% and matrix category about 3.0%. This pattern indicates that matrix penalties scale primarily with inherent recoverability and structure and are then modified by the acid–base form present at the applied loading pH and by the matrix composition.^76^ Strong contributions from the pH and matrix descriptors agree with evidence that pH-dependent protonation and matrix-driven ionisation effects can markedly alter sorption and apparent recovery in liquid chromatography with electrospray detection.^75^ Consequently, the mechanistic interpretation here emphasises that analyte identity sets the recoverability ceiling, pH selects the dominant ionisation state during loading, and matrix category approximates the direction and magnitude of competitive and ionisation effects when direct chemistry measurements are unreported.^75,76^

The attribution for the enrichment factor and detection and quantification limits under ElasticNet is dominated by engineered response scale variables, such as log enrichment +1, log LOD, and log LOQ, with other predictors contributing only minor average changes in performance. This dominance suggests that the linear sensitivity models primarily encode response scale, location, and shared reporting structure rather than distinct process-level mechanisms.^13,77^ From a method development standpoint, the joint Shapley and permutation profiles, therefore, support a mechanistic narrative in which the loading pH selects ionisation states, total mass and sorbent chemistry set the balance between capacity and affinity, eluent volume governs desorption, and matrix category stands in for ionic strength and co extracted organic matter, all interpreted alongside study-level structure to inform robust optimisation rather than overconfident point prediction.^77^

### 3.6 Error landscapes and operating recommendations

**Fig. 6:**
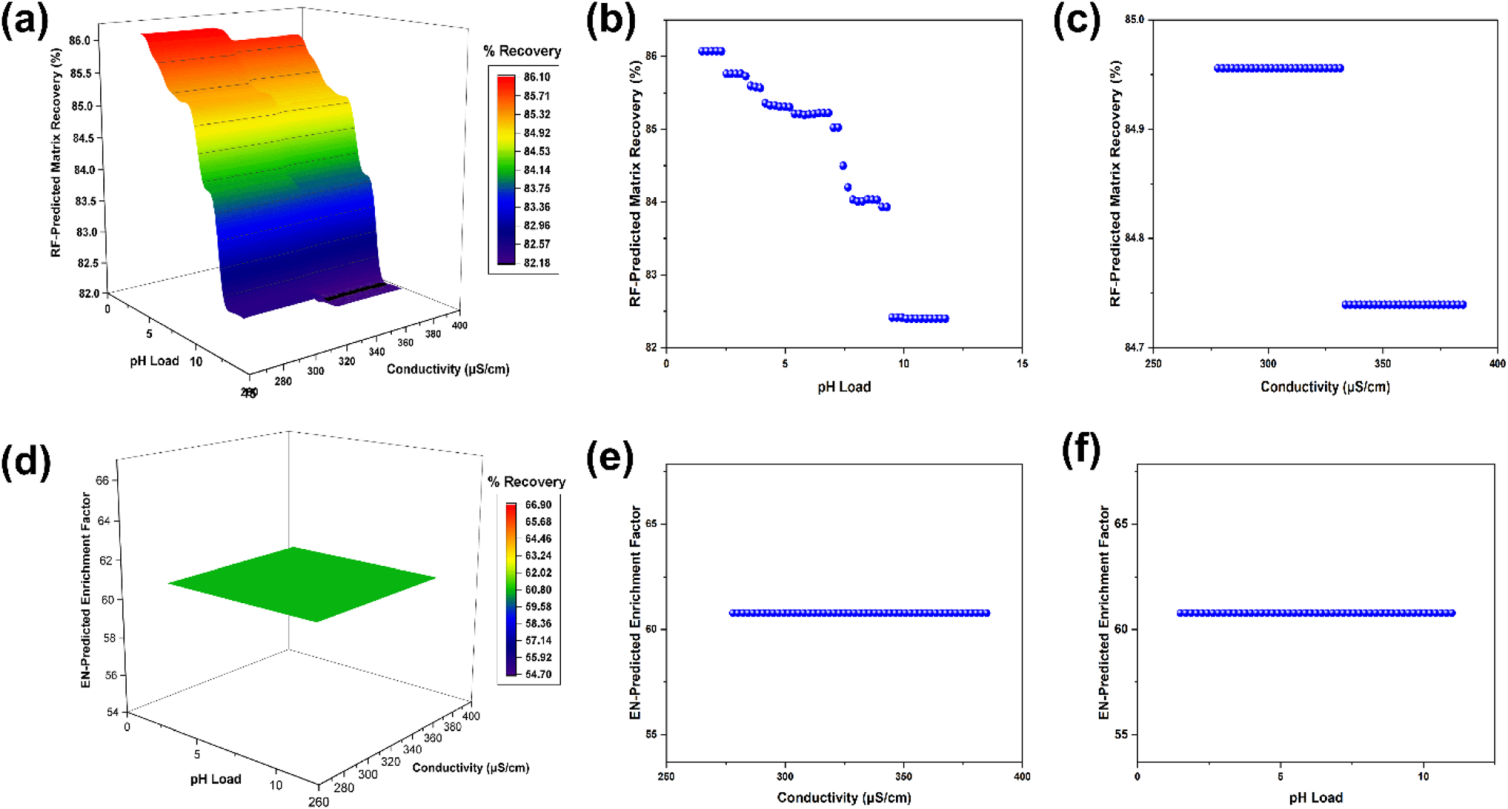
Error surfaces across pH and conductivity and error *versus* feature profiles: (a) three-dimensional surface or filled contour of error across the pH and conductivity grid for matrix recovery. (b) One-dimensional plot for recovery *vs.* pH load and (c) one-dimensional plot for recovery *vs.* conductivity. (d) Three-dimensional surface or filled contour of error across the pH and conductivity grid for the enrichment factor. (e) One-dimensional plot for the enrichment factor *vs.* conductivity and (f) one-dimensional plot for the enrichment factor *vs.* pH load.

Three-dimensional surfaces across the pH and conductivity grid illustrated in Fig. 6 confirmed that only the recovery outcome yielded a populated landscape suitable for interpretation, with the surface for the recovery matrix (%) using the random forest champion showing a contiguous basin (Fig. 6a) rather than isolated minima. Across the grid, predicted recovery varied by about 4 %pt, from 82.20 at the alkaline edge of the grid to 86.08 under more acidic conditions, with the shallow optimum located near a pH of approximately 2 and the lower end of the observed conductivity range around 280 µS cm^−1^, indicating a robust region with limited sensitivity to moderate ionic strength variation. The one-dimensional profiles are consistent with the surface: the pH partial dependence for the recovery matrix (%) spans 82.40 to 86.07 across the evaluated range (Fig. 6b), while the conductivity profile is nearly flat over 278 to 385 µS cm^−1^, changing by less than 0.2 %pt (Fig. 6c). The matrix recovery ratio exhibits a similar qualitative pattern, with the pH profile rising from 86.37 to 90.85 and peaking near a pH of about 5.75, before declining toward the alkaline boundary, a trajectory that aligns with the expected interplay between analyte ionisation and sorbent charge domains during load and wash. In contrast, the enrichment factor and detection targets display essentially flat partial dependence across the pH and conductivity, as shown in Fig. 6d. This implies a limited leverage of these axes within the sampled operating space and shifting the emphasis toward other factors, such as eluent composition and volume, that govern desorption strength. This is also confirmed by the linear plot for conductivity (Fig. 6e) and pH load (Fig. 6f).

Taken together, the landscapes support practical recommendations that privilege mildly acidic loading conditions with low to moderate conductivity as a default operating band for robust recovery while avoiding strongly alkaline loads that coincide with the elevated error on the grid for the primary target set. This guidance is consistent with sample preparation literature in which pH adjustment modulates ionisation and hydrophobic interactions in solid-phase extraction and related formats, whereas moderate ionic strength primarily affects the co-extraction of dissolved organic matter rather than directing primary retention, especially under reversed phase or mixed mode mechanisms.^78^ Additional reassurance comes from large-volume liquid chromatography studies reporting stable quantitative performance across moderate salinity windows when extraction chemistry is appropriately matched to analyte acid–base properties, echoing the flat conductivity profiles observed here.^79^

### 3.7 Hyperparameter landscapes and robustness

Hyperparameter landscapes were interrogated to demonstrate that the selected models are supported by stable basins of near-optimal performance and to quantify sensitivity to small movements in dominant hyperparameters. This diagnostic is warranted because cross-validation protocols that tune multiple configurations and report the best resampled score can yield optimistic generalisation estimates and unstable selections in finite samples.^80^ Estimation noise is further amplified in high-dimensional, correlated descriptor spaces, where apparent optima can be artefacts of partitioning rather than durable signals.^80,81^ Consequently, robustness checks are essential whenever tuning and any data-dependent preprocessing are coupled to the same resampling design, to minimise leakage and selection bias.^82^ Recent guidance for environmental machine learning similarly emphasises the transparent reporting of tuning strategy and robustness diagnostics as a prerequisite for credible and reusable modelling claims.^83^

**Fig. 7:**
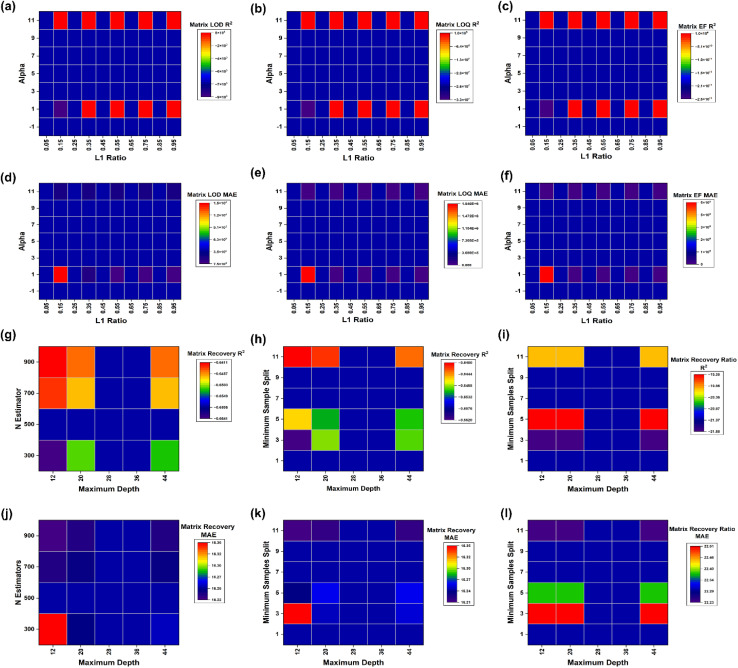
Hyperparameter heatmaps for ElasticNet and random forest models. (a) Matrix LOD *R*^2^ across ElasticNet alpha and L1 ratio, (b) matrix LOQ *R*^2^ across ElasticNet alpha and L1 ratio, (c) enrichment factor *R*^2^ across ElasticNet alpha and L1 ratio, (d) matrix LOD MAE across ElasticNet alpha and L1 ratio, (e) matrix LOQ MAE across ElasticNet alpha and L1 ratio, (f) enrichment factor MAE across ElasticNet alpha and L1 ratio, (g) matrix recovery *R*^2^ across random forest *n* estimators and maximum depth, (h) matrix recovery *R*^2^ across random forest minimum samples split and maximum depth, (i) matrix recovery ratio *R*^2^ across random forest minimum samples split and maximum depth, (j) matrix recovery MAE across random forest *n* estimators and maximum depth, (k) matrix recovery MAE across random forest minimum samples split and maximum depth, and (l) matrix recovery ratio MAE across random forest minimum samples split and maximum depth.

**Fig. 8:**
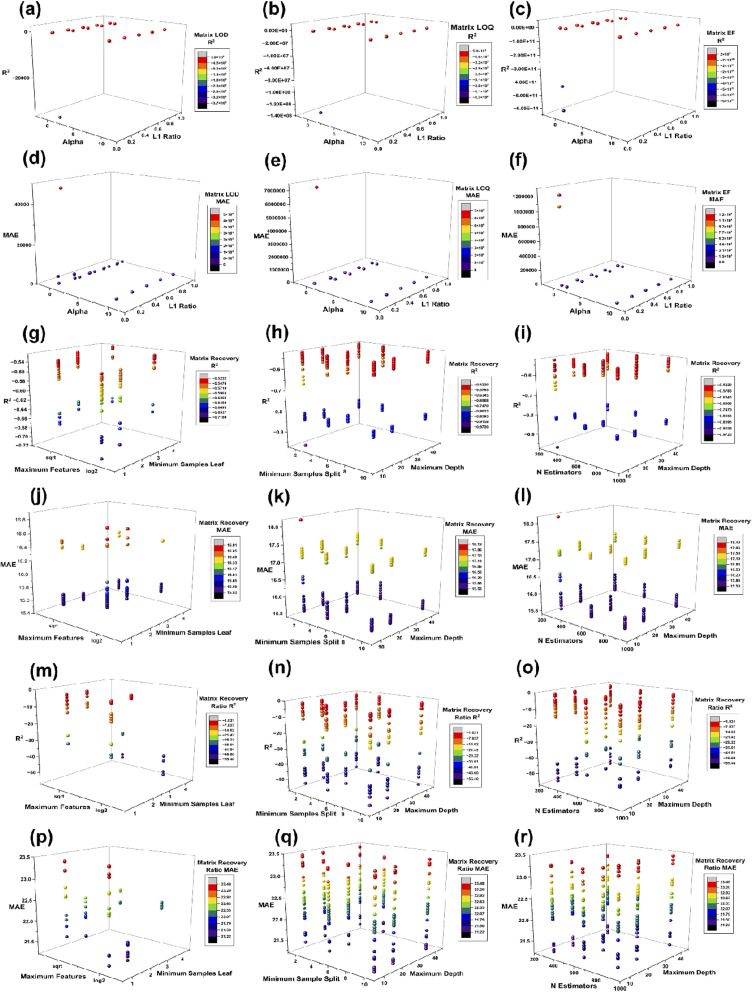
Three-dimensional hyperparameter landscapes for ElasticNet and random forest models. (a) Matrix LOD *R*^2^ across ElasticNet alpha and L1 ratio, (b) Matrix LOQ *R*^2^ across ElasticNet alpha and L1 ratio, (c) enrichment factor *R*^2^ across ElasticNet alpha and L1 ratio, (d) matrix LOD MAE across ElasticNet alpha and L1 ratio, (e) matrix LOQ MAE across ElasticNet alpha and L1 ratio, (f) enrichment factor MAE across ElasticNet alpha and L1 ratio, (g) matrix recovery *R*^2^ across random forest maximum features and minimum samples leaf, (h) matrix recovery *R*^2^ across random forest minimum samples split and maximum depth, (i) matrix recovery *R*^2^ across random forest *n* estimators and maximum depth, (j) matrix recovery MAE across random forest maximum features and minimum samples leaf, (k) matrix recovery MAE across random forest minimum samples split and maximum depth, (l) matrix recovery MAE across random forest *n* estimators and maximum depth, (m) matrix recovery ratio *R*^2^ across random forest maximum features and minimum samples leaf, (n) matrix recovery ratio *R*^2^ across random forest minimum samples split and maximum depth, (o) matrix recovery ratio *R*^2^ across random forest *n* estimators and maximum depth, (p) matrix recovery ratio MAE across random forest maximum features and minimum samples leaf, (q) matrix recovery ratio MAE across random forest minimum samples split and maximum depth, and (r) matrix recovery ratio MAE across random forest *n* estimators and maximum depth.

For ElasticNet sensitivity endpoints, the *R*^2^ heatmaps for matrix LOD, matrix LOQ, enrichment factor (Fig. 7a–c), as well as the companion MAE heatmaps (Fig. 7d–f) show a ridge-like corridor of high skill anchored at a high L1 ratio, with a pronounced deterioration at the lowest L1 ratio values across endpoints. The three-dimensional panels confirm that high skill points form a continuous ridge rather than an isolated spike, for *R*^2^ in Fig. 8a–c and MAE in Fig. 8d and e, providing direct reassurance that the selected settings are not single-point peaks. This behaviour is consistent with the role of the penalty mixture in stabilising coefficient estimation under correlated predictors, where stronger L1 components promote sparsity while retaining the grouped selection *via* the L2 component.^43^ The table reinforces the basin structure. For the enrichment factor, the top five configurations cluster at alpha 0.001 with an L1 ratio between 0.1 and 0.9, delivering *R*^2^ means of about 1.000, RMSE means from 0.309 to 3.044, and MAE means from 0.110 to 1.084. For the matrix LOD, the leading configuration at alpha 0.001 and an L1 ratio of 0.9 yields a *R*^2^ mean of 1.000, with a RMSE mean of 29.508 and a MAE mean of 9.675, while neighbouring ridge settings retain high *R*^2^ means between 0.966 and 0.993. For the matrix LOQ, the best configuration at alpha 0.01 and an L1 ratio of 0.9 achieves a *R*^2^ mean of 0.998, a RMSE mean of 790.989 and a MAE mean of 256.421, while alpha 0.001 and the L1 ratio of 0.9 remain competitive at a *R*^2^ mean of 0.988 with a lower MAE mean of 211.556, indicating a modest bias variance tradeoff rather than sharp instability.

The random forest landscapes for matrix recovery and matrix recovery ratio are comparatively flat, indicating limited hyperparameter leverage relative to residual heterogeneity. Prior work shows that random forest performance often saturates across wide ranges of tree counts and splitting controls, with most gains concentrated in avoiding extreme complexity or overly restrictive node constraints.^84^ In this study, recovery *R*^2^ heatmaps (Fig. 7g and h) and recovery ratio *R*^2^ heatmaps (Fig. 7i) show only modest gradients across maximum depth, minimum samples split, and related controls, while MAE panels (Fig. 7j–l) remain similarly compressed. The three-dimensional plots provide the same message, with recovery *R*^2^ and recovery ratio *R*^2^ shown in Fig. 8g–I and m–o, and their corresponding MAE structures shown in Fig. 8g–l and p–r, respectively, showing broad plateaus rather than narrow optima. Table S6 quantifies these plateaus. The best five recovery configurations cluster tightly around *n* estimators = 900, max features set to sqrt, and minimum samples leaf = 4, yielding *R*^2^ means around −0.5233, with RMSE means between 21.216 and 21.226 and MAE means between 15.532 and 15.537. For the matrix recovery ratio, the top four configurations are effectively identical at a *R*^2^ mean of −1.031 with a RMSE mean of 27.108 and MAE mean of 21.389, followed by a clear drop in the fifth configuration, consistent with a flat basin punctuated by worse regions rather than peak picking noise. The limited hyperparameter leverage is also scientifically plausible because recovery and matrix effects in complex waters can be dominated by unmeasured co-extractives and ionisation behaviour, constraining predictable variance, regardless of learner choice.^4,27^

Collectively, Fig. 7, 8 and Table S6 show that the final hyperparameter selections are supported by stability basins, strengthening confidence that downstream interpretations reflect data structure rather than tuning artefacts. This emphasis on robustness aligns with evidence that validation design, including external checks where feasible, is often more consequential than marginal hyperparameter refinements once sensible ranges are reached.^85^

### 3.8 Calibration and predictive intervals

**Fig. 9:**
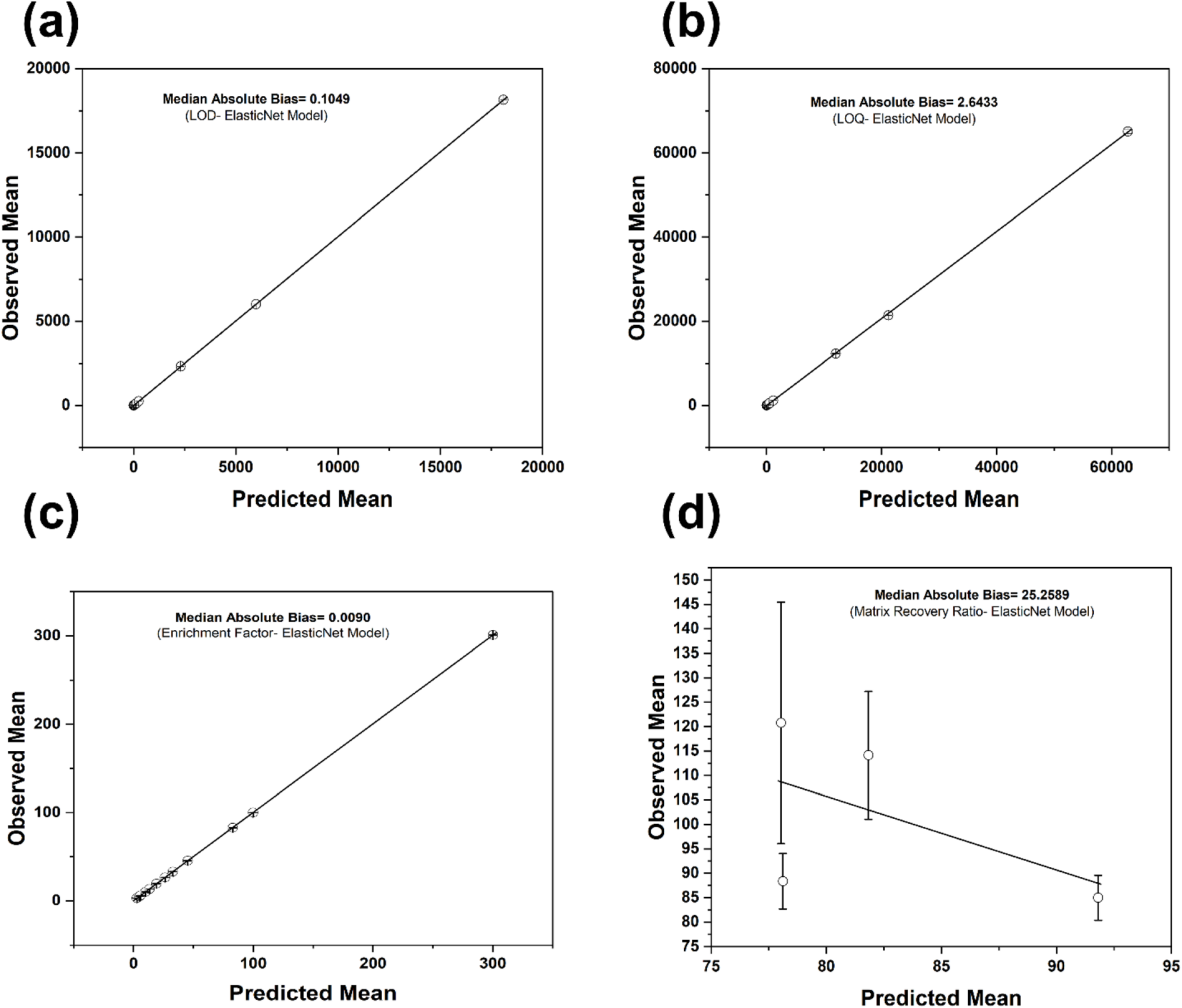
Reliability curve for the different targets. Bin-wise observed *versus* predicted means with diagonal reference and standard error bars for the (a) LOD, (b) LOQ, (c) enrichment factor, and (d) matrix recovery ratio.

Reliability analysis based on binned observed and predicted means showed that the three sensitivity targets were well calibrated in central tendency, whereas the matrix recovery ratio was not (see details in Fig. 9). For the enrichment factor, LOD and LOQ, the reliability curves close to the diagonal- and bin-specific deviations were small in the modelling scale, indicating accurate magnitude calibration over most of the prediction range. The median absolute bias across bins was about 0.10 for the LOD (Fig. 9a), 2.64 for the LOQ (Fig. 9b) and 0.009 for the enrichment factor (Fig. 9c) but around 25.26 for the matrix recovery ratio, indicating residual structure beyond the modelled covariates (see Fig. 9d). These findings agree with studies that treat reliability diagrams as a primary calibration diagnostic in regression and recommend reporting them together with error summaries when models support applied prediction and method optimisation.^86^

For the ElasticNet champions, conformal prediction intervals for the sensitivity endpoints achieved coverage close to nominal while remaining comparatively narrow. At the 90% level, empirical coverage was about 0.91 for the LOD and LOQ and 0.92 for the enrichment factor, with mean widths of roughly 44 ng L^−1^ for the LOD, 830 ng L^−1^ for the LOQ and 0.30 in the modelled scale for the enrichment factor. At 95%, coverage increased to around 0.95 to 0.96 with corresponding widths near 65 ng L^−1^, 1750 ng L^−1^ and 0.84, respectively. Fold-wise coverage was stable across splits, apart from one LOQ fold with clear under-coverage, showing that interval behaviour is still influenced by the distribution of studies and matrices in each partition. The combination of near nominal coverage and target-specific widths matches reports from conformal prediction in chemometric and materials applications, where calibrated intervals typically attain nominal coverage while widening in regions with higher response variance.^87^

The calibration for the matrix recovery ratio using random forest and conformal intervals was substantially weaker. Empirical coverage was 0.651 at the 90% setting and 0.663 at 95%, despite the mean interval widths above 70 %pt. This is well detailed in Table 6. Fold-wise coverage clustered around 0.78 at both levels, so the poorest calibrated fold dominated the pooled coverage once all held-out cases were combined. Isotonic calibration maps compressed predictions towards approximately 90% with slopes close to zero, which indicates that the underlying point predictions offered limited resolution across the range of recovery ratios even after monotone recalibration. This behaviour is consistent with matrix effect studies in quantitative liquid chromatography with mass spectrometric detection, where unmeasured matrix chemistry and co-extracted constituents have been shown to perturb apparent recoveries and limit attainable calibration when descriptors for these factors are incomplete.^27^

**Table 6:** Interval coverage and width revealing fold-wise and overall coverage at 90% and 95% nominal levels with mean prediction-interval width for champion models

Target	Model	Nominal coverage	Folds	Held-out cases (*n*)	Fold coverage median (min–max)	Empirical coverage overall	Mean interval width
Matrix recovery ratio	Random forest regressor	0.95	3	196	0.767 (0.593–1.000)	0.663	84.781
Matrix recovery ratio	Random forest regressor	0.90	3	196	0.733 (0.593–1.000)	0.651	74.847
Enrichment factor	ElasticNet	0.95	5	1313	0.963 (0.897–1.000)	0.956	0.841
Enrichment factor	ElasticNet	0.90	5	1313	0.929 (0.897–1.000)	0.922	0.300
LOD matrix (ng L^−1^)	ElasticNet	0.95	5	1140	0.956 (0.908–1.000)	0.952	64.707
LOD matrix (ng L^−1^)	ElasticNet	0.90	5	1140	0.956 (0.829–1.000)	0.912	44.386
LOQ matrix (ng L^−1^)	ElasticNet	0.95	5	1010	1.000 (0.827–1.000)	0.959	1754.599
LOQ matrix (ng L^−1^)	ElasticNet	0.90	5	1010	1.000 (0.618–1.000)	0.906	829.752

Row-wise prediction intervals further emphasised the contrast between sensitivity and recovery targets detailed in Table 7. For the enrichment factor, intervals centred near 15 had widths of about 0.3 at 90% and 0.84 at 95%, indicating tight uncertainty control around the ElasticNet predictions. For the LOD, intervals around 100 ng L^−1^ typically spanned only tens of nanograms, whereas LOQ intervals for difficult matrices extended from 0 to beyond 1400 ng L^−1^, reflecting the high variance of that endpoint. For the matrix recovery ratio, per case intervals of roughly 70 to 90 %pt were still insufficient to restore nominal coverage, mirroring the summary under coverage and the isotonic maps. Together, the calibration results support the use of conformal intervals for the LOD, LOQ and enrichment factor when planning method margins and comparing candidate conditions. The matrix recovery ratio requires additional matrix-aware covariates or more aggressive post calibration before it can underpin interval-based compliance decisions.^87^

**Table 7:** Per-row prediction intervals (illustrative subset) showing lower and upper bounds for 95% and 90% conformal intervals for representative held-out cases, with study and analyte identifiers for champion models

Target	Model	DOI	Study ID	Analyte	Matrix category	*y* (true)	*ŷ* (pred)	95% lower	95% upper	Covered 95%	Width 95%	90% lower	90% upper	Covered 90%	Width 90%
Matrix recovery ratio	Random forest regressor	10.1002/jssc.202200042	Li_2022_PAIL_CFP	Tolmetin	Ultrapure	76.80	78.114	9.344	100.000	1	90.656	19.277	100.000	1	80.723
Enrichment factor	ElasticNet	10.1016/j.microc.2021.106798	Shahriman_2021_PIL_pTFME	Sulfadiazine	Ultrapure	14.99	14.999	14.579	15.420	1	0.841	14.849	15.149	1	0.300
LOD matrix (ngL^−1^)	ElasticNet	10.1016/j.talanta.2022.124188	Shahriman_2023_pTFME_PolyMMAIL_FP	Oxytetracycline	River	110.00	109.882	71.549	148.215	1	76.666	84.427	135.338	1	50.911
LOQ matrix (ngL^−1^)	ElasticNet	10.1016/j.microc.2024.110355	Olorunnisola_2024_CMDI1_SPE	Chloramphenicol	Tap	217.70	218.107	0.000	1479.921	1	1479.921	0.000	768.638	1	768.638

### 3.9 Ablation and preprocessing sensitivity

**Fig. 10:**
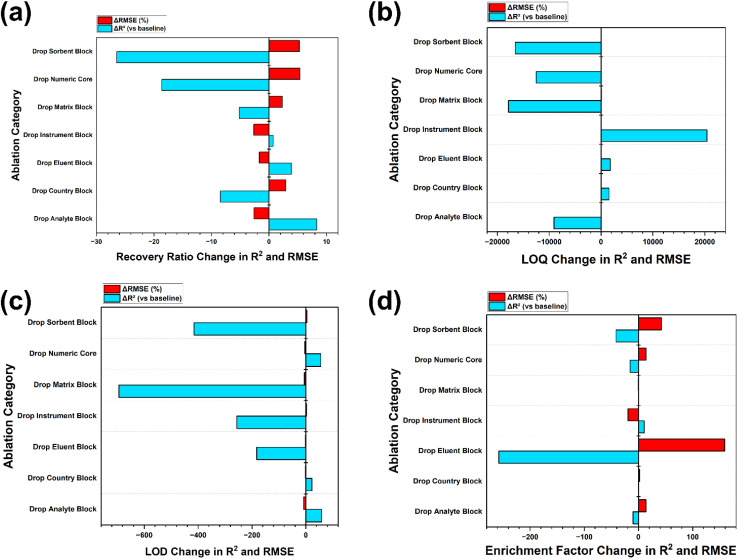
Ablation bars for each target showing the change in *R*^2^ and percentage change in RMSE when removing each variable family: (a) matrix recovery ratio, (b) LOQ, (c) LOD, and (d) enrichment factor.

Ablations were performed to quantify the contribution of each variable family. For the recovery in matrix, the random forest baseline achieved *R*^2^ near −0.14 with a RMSE around 19 %pt. Removing matrix descriptors had the strongest effect, lowering the *R*^2^ by about 0.09 and increasing the RMSE by almost 3%. Dropping instrument descriptors produced a slightly smaller loss of about 0.08 in the *R*^2^ and a rise in RMSE of just under 2%. Other blocks caused only modest changes, and the removal of eluent descriptors marginally reduced the RMSE, indicating limited unique information once the matrix, instrument, and numeric core fields were present. For the matrix to ultrapure recovery ratio illustrated in Fig. 10a, sorbent descriptors were most critical. Baseline performance already showed a negative *R*^2^ and a RMSE near 26 points, and the ablation of sorbent descriptors decreased *R*^2^ by about 26 points and increased the RMSE by just over 5%, as detailed in Table S7. Removing numeric core fields caused the second largest deterioration, with an 18-point loss in *R*^2^ and a similar 5% rise in RMSE, whereas the removal of matrix or country descriptors had more moderate impacts, and the ablation of analyte or eluent descriptors slightly improved the average error, consistent with partial redundancy in those families.

ElasticNet ablations enlisted in Table S8 for the sensitivity endpoints highlighted related target-specific levers. For the limits of quantification plotted in Fig. 10b, matrix descriptors were indispensable: removing the matrix block increased the RMSE from around 37 000 to more than 60 000 ng L^−1^, a rise of roughly 62%, while ablations of the analyte, numeric core, or instrument fields all reduced the RMSE modestly. The limits of detection were more robust, with only sorbent and instrument ablations producing noticeable RMSE increases of about 4 and 3% (Fig. 10c), respectively. For the enrichment factor, eluent descriptors dominated, since their removal increased the RMSE by approximately 160%, and the removal of sorbent descriptors raised the RMSE by more than 40%, whereas matrix removal had a negligible impact, and instrument removal slightly improved the fit (see Fig. 10d).

These patterns echo experimental work in liquid chromatography that attributes much variability in matrix effects and extraction efficiency to matrix composition, instrument configuration, and sorbent chemistry.^88^ Quantitative validation studies show that the control of the matrix type, sorbent functionality, and eluent strength reduces signal suppression and improves detection and quantification limits.^60,88^ Machine learning applications to liquid chromatography and mass spectrometry increasingly use the grouped feature selection or ablation to reveal dominant feature blocks without a loss in accuracy, which is consistent with the concentrated impact of the matrix, sorbent, numeric core, and eluent families observed here.^89^

### 3.10 Practical recommendations and decision support

**Fig. 11:**
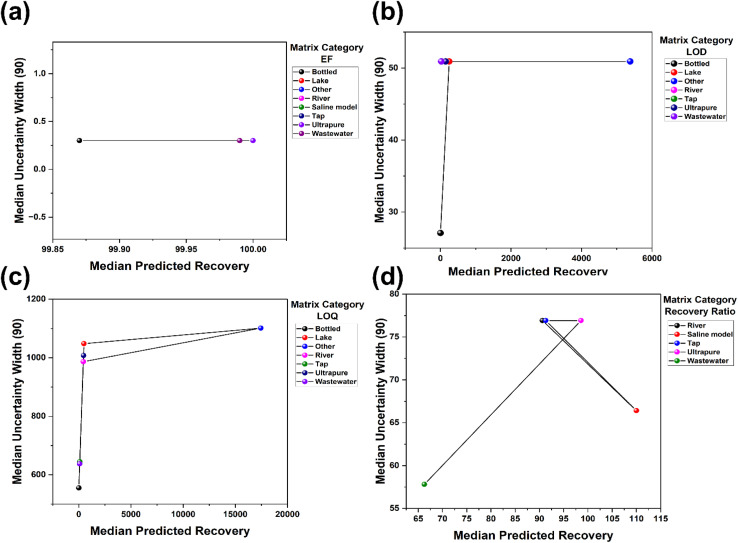
Decision map showing predicted recovery *versus* uncertainty width for representative settings to guide tradeoffs for the (a) enrichment factor, (b) limit of detection, (c) limit of quantification, and (d) matrix recovery ratio.

Quantitative outputs across endpoints provide a structured basis for the operating guidance in this multi-study cellulose-based extraction dataset. Table 8 links the champion manifest to calibration summaries and error by group metrics and shows that ElasticNet is retained for the enrichment factor and for both detection limits, whereas random forest remains preferred for the recovery in matrix and matrix to ultrapure recovery ratio. Model comparison by fold indicates that pair-wise Wilcoxon tests between ElasticNet, random forest, and gradient boosted trees seldom yield adjusted *p*-values below conventional thresholds, so champion choices rest on modest gains in average error, interval behaviour, and interpretability rather than strict significance. For the enrichment factor, conformal prediction intervals achieve empirical coverage close to nominal at 90 and 95% with mean widths near 0.3 and 0.8 in the model scale, with a RMSE below one unit across matrices. This supports their use when planning pre-concentration factors, as illustrated in Fig. 11a. For the limits of detection plotted in Fig. 11b, coverage is similarly close to nominal, with mean interval widths around 45 and 65 ng L^−1^. The limits of quantification retain the widest intervals and strong matrix dependence (see Fig. 11c), while the matrix recovery ratio exhibits under-coverage and recovery RMSE above 30 %pt in wastewater, so ratio predictions are best interpreted as conservative risk flags that complement rather than replace experimental recovery measurements (Fig. 11d).

**Table 8:** Suggested operating bands by matrix category with supporting recovery error and 90% interval characteristics for champion models

Matrix category	RMSE recovery matrix (%)	Coverage 90 enrichment factor	Width 90 enrichment factor	Coverage 90 LOD matrix (ng L^−1^)	Width 90 LOD matrix (ng L^−1^)	Coverage 90 LOQ matrix (ng L^−1^)	Width 90 LOQ matrix (ng L^−1^)	Coverage 90 matrix recovery ratio	Width 90 matrix recovery ratio
Bottled	12.2	1.000	0.30	1.000	26.79	1.000	554.56	—	—
Lake	15.1	1.000	0.30	0.868	47.42	0.897	927.82	—	—
Other	9.1	1.000	0.30	0.615	50.91	0.615	1066.56	—	—
River	16.9	0.932	0.30	0.873	44.40	0.884	881.74	0.593	78.21
Saline model	—	1.000	0.30	—	—	—	—	1.000	66.41
Tap	18.5	0.966	0.30	1.000	43.85	1.000	774.11	0.593	78.21
Ultrapure	11.8	0.946	0.30	1.000	49.54	0.991	932.23	0.607	77.79
Wastewater	31.4	0.870	0.30	0.974	44.65	1.000	622.69	0.733	57.82

The decision map shown in Fig. 11 converts these summaries into a practical tool by plotting the predicted means against interval widths for representative combinations of the loading pH, matrix class, sorbent chemistry, and eluent settings. This data is also detailed in Table S9. Analysts can prioritise configurations that combine adequate predicted recovery or sensitivity with intervals that are narrow enough for the intended regulatory or screening decision and can avoid settings that yield low expected performance or very wide uncertainty bands. Surfaces of error in pH and conductivity, together with the ablation experiments, indicate that recovery errors are smallest near neutral pH levels with moderate conductivity and increase at extreme pH levels or under highly conductive conditions. They also indicate that matrix descriptors and sorbent chemistry are indispensable for the recovery ratio and that eluent descriptors dominate enrichment factor performance. Error by group diagnostics further show that wastewater carries the largest uncertainty, with a recovery root mean square error exceeding 30%p, and detection and quantification intervals that widen markedly relative to river, tap, and bottled water, so predictions in complex matrices should be treated as conservative lower bound scenarios that always require local verification.

These quantitative patterns support concrete recommendations for future study design and reporting. A minimum metadata set should include explicit matrix category; sample pH at load; bulk conductivity; at least one proxy for dissolved organic carbon; core analyte descriptors, such as class and charge at environmental pH; and succinct descriptors of sorbent chemistry and eluent composition, so that future models can represent matrix effects and mechanistic drivers more completely. Evidence from recent assessments of the matrix effect in quantitative liquid chromatography tandem mass spectrometry highlights the importance of systematic matrix characterisation and documentation of sample pH, ionic strength, and organic content for transferable calibration and bias control.^27,90^ Complementary reviews and optimisation studies for solid-phase extraction identify loading pH, eluent type, and volume, and sorbent chemistry as primary levers for recovery and matrix effect mitigation, closely matching the variable families with the largest ablation impact in this study.^91^ Applications of conformal prediction in chemometrics and quantitative structure activity modelling show that calibrated prediction intervals can routinely achieve near nominal coverage without strong distributional assumptions, supporting their use as a standard component of machine learning-assisted method development and decision support.^87,92^

## 4 Limitations and future work

Although the present study delivers a reproducible machine learning framework for quantifying cellulose-based solid-phase extraction performance for pharmaceuticals in water, several limitations constrain the generality and strength of inference about matrix effects. The evidence base is restricted to peer-reviewed English language studies published from 2015 to 2025. Therefore, grey literature, earlier validation work, non-english protocols, and technical reports that could broaden the matrix coverage and reveal negative or null outcomes are not represented, which can inflate apparent success rates and narrow the observed operating space. The curated dataset remains modest in size, with imbalance across matrices and endpoints, as well as sparse reporting of key matrix chemistry covariates, such as dissolved organic carbon, conductivity-linked ionic strength, UV_254_ proxies, and co-extracted organic matter, which are recognised drivers of recovery variability and ion suppression in complex extracts.^19,93,94^ In this setting, negative *R*^2^ behaviour and wide prediction intervals for recovery are interpreted as evidence-based limited predictability dominated by missing variable bias and heterogeneous reporting, rather than a failure of the learning algorithms.^6,64^

Further limitation arises from the inability to apply defensible study quality weighting because the reporting of ISO-aligned validation elements, quality control procedures, sample size adequacy, and analytical uncertainty is inconsistent across the existing literature, making objective weights difficult without introducing subjective scoring. The eligibility scope prioritises end-to-end extraction workflows, so adsorption-only studies were excluded from quantitative modelling to preserve endpoint comparability, although adsorption-focused evidence remains mechanistically valuable and should be integrated in the future through linked mechanistic descriptors and hierarchical evidence structures.^19^ Future work should expand the evidence base through targeted experimental campaigns that systematically vary matrix chemistry and operational windows, with minimum reporting standards for conductivity, dissolved organic carbon, UV_254_, major ions, and salinity explicitly enforced to strengthen transportability.^94^ Incorporating richer sorbent and analyte descriptors, including surface chemistry, pore architecture, and quantum chemically derived interaction indices, would align the approach with advances in adsorption modelling that improve interpretability.^12–14^ Prospective validation in independent laboratories and matrices, coupled with covariate-shift-aware conformal prediction, is required to move from retrospective diagnosis to actionable decision support for method transfer, sorbent selection, and quality assurance across monitoring networks.^95^

## 5 Conclusion

This study establishes an auditable machine learning framework for quantifying cellulose-based solid-phase extraction performance for pharmaceuticals in aqueous matrices. A curated dataset of primary studies from 2015 to 2025 was harmonised into a common variable dictionary that captures controllable method settings, analyte descriptors, and reported matrix covariates, alongside recovery, enrichment, and sensitivity endpoints. Nested grouped cross-validation by study, coupled with systematic hyperparameter exploration, mixed effects models, conformal prediction, and ablation experiments, was used to characterise predictive skill, transportability, and calibration while preserving study identity and limiting optimistic leakage.

Quantitatively, the enrichment factor and limits of detection and quantification proved highly predictable from the reported method variables, with linear ElasticNet models delivering near-perfect explained variance and narrow absolute errors under out-of-study evaluation. In contrast, the recovery in matrix, and especially the matrix to ultrapure recovery ratio, remained only weakly predictable across studies, with large residuals in wastewater and river categories and wide, variably covered intervals even after isotonic calibration. Negative *R*^2^ behaviour for recovery should be interpreted as evidence-based bounded predictability driven primarily by heterogeneous reporting and missing matrix chemistry drivers, rather than as a failure of the modelling strategy. Error discovered across pH and the available conductivity fields identified a shallow operating basin for recovery, while leaving one study out, diagnostics indicated that part of the remaining variability reflects latent protocol differences that are not fully captured by published descriptors. These findings indicate that enrichment and sensitivity can be planned reliably from existing validation practice. However, the recovery and ratio behaviour still require local measurements and systematic reporting of matrix chemistry, including conductivity, dissolved organic carbon, UV_254_ proxies, and major ions, to improve transportability. By making the data, workflow, and figures transparent, this framework provides a practical decision support and reporting audit tool and can be extended to other sorbent families once comparable descriptor coverage is available.

## Author contributions

E. A: conceptualization, data curation, formal analysis, writing – original draft, writing – review & editing; D. O: writing – original draft, writing – review & editing; M. O. A: writing – original draft, writing – review & editing; O. E: writing – original draft, writing – review & editing; and M. O. O: conceptualization, data curation, supervision, validation, visualization, writing – original draft, writing – review & editing.

## Conflicts of interest

There are no conflicts to declare.

## Data Availability

No primary research results, software or codes have been included, and no new data were generated or analysed as part of this review paper.
